# Higher diet quality is associated with short and long-term benefits on SF-6D health state utilities: a 5-year cohort study in an international sample of people with multiple sclerosis

**DOI:** 10.1007/s11136-023-03361-w

**Published:** 2023-02-23

**Authors:** Harry Kirkland, Julie Campbell, Jeanette Reece, Nupur Nag, Yasmine Probst, Sandra Neate, Alysha De Livera, George Jelinek, Steve Simpson-Yap

**Affiliations:** 1 Neuroepidemiology Unit, Melbourne School of Population & Global Health, The University of Melbourne, Melbourne, Australia; 2 Menzies Institute for Medical Research, University of Tasmania, Hobart, Australia; 3 School of Medical, Indigenous and Health Sciences, Illawarra Health and Medical Research Institute, University of Wollongong, Wollongong, Australia; 4 Baker Heart and Diabetes Research Institute, Melbourne, Australia; 5 Mathematics and Statistics, School of Computing, Engineering and Mathematical Sciences, La Trobe University, Melbourne, Australia

**Keywords:** Multiple sclerosis, Health state utility, Diet, Epidemiology

## Abstract

**Background/Purpose:**

Health state utilities (HSU) are a subjective measure of an individual's health-related quality of life (HRQoL), adjusted by societal or patient relative preference weights for living in different states of health-related quality of life (HRQoL), derived from patient-reported responses to multi-attribute utility instrument (MAUI), and can be used as inputs for cost-utility analyses and in clinical assessment. This research assessed associations of diet with subsequent HSU in a large international cohort of people living with multiple sclerosis (MS), a progressive autoimmune condition of the central nervous system.

**Methods:**

HSUs were generated from responses to Short-Form Six-Dimension (SF-6D) MAUI, and quality-of-the-diet by Diet Habits Questionnaire (DHQ). Cross-sectional, and short- and long-term prospective associations of DHQ with HSU evaluated by linear regression at 2.5- and 5-years. Pooled prospective associations between DHQ and HSU evaluated using linear and quantile regression. Analyses adjusted for relevant demographic and clinical covariates.

**Results:**

Among 839 participants, baseline DHQ scores showed short- and long-term associations with subsequent HSU, each 10-unit increase in total DHQ score associated with 0.008–0.012 higher HSU (out of 1.00). These associations were dose-dependent, those in the top two quartiles of baseline DHQ scores having 0.01–0.03 higher HSU at follow-up, 0.03 being the threshold for a minimally clinically important difference. Fat, fiber, and fruit/vegetable DHQ subscores were most strongly and consistently associated with better HSU outcomes. However, baseline meat and dairy consumption were associated with 0.01–0.02 lower HSU at subsequent follow-up.

**Conclusions:**

A higher quality-of-the-diet showed robust prospective relationships with higher HSUs 2.5- and 5-years later, substantiating previous cross-sectional relationships in this cohort. Subject to replication, these results suggest interventions to improve the quality-of-the-diet may be effective to improve HRQoL in people living with MS.

**Supplementary Information:**

The online version contains supplementary material available at 10.1007/s11136-023-03361-w.

## Introduction

Multiple sclerosis (MS) is a chronic inflammatory and neurodegenerative autoimmune condition of the central nervous system, characterised by axonal injury and demyelination [1]. The progressive loss of motor function, alongside other clinical manifestations such as fatigue and depression [2–5], can have a substantial effect on physical and/or psychosocial aspects of wellbeing, leading to reduced health-related quality of life (HRQoL) for people with MS (pwMS) [6, 7]. Multiple modifiable lifestyle behaviours have been associated with MS progression and decreased HRQoL in pwMS, including negative impacts from smoking and BMI, and positive effects of physical activity and meditation [8, 9]. Accumulating evidence has suggested beneficial relationships between healthier quality-of-diet and MS onset and disease progression [10], including relapse rate, disability progression, and depression [11–15].

Health state utilities (HSU) are a subjective measure of an individual's HRQoL, adjusted by societal or patient relative preference weights for living in different states of health-related quality of life (HRQoL), derived from patient-reported responses to a multi-attribute utility instrument’s (MAUI) questionnaire (e.g., SF-6D, EQ-5D-5L, EQ-5D-5L-Psychosocial, or AQoL-8D MAUIs [16–19]) about symptoms, functional limitations, and overall functions relating to HRQoL [20]. The derived HSU is a measure between ‘0’ and ‘1’, with 0 representing “death” and 1 representing full health. Importantly, HSUs are an input metric to quality-adjusted life years (QALYs) in cost-utility analysis, both critical to translation to practice [21]. HSUs are used in clinical practice settings and as predictors of health [22–25]. Most MAUIs have country-specific population norms and minimal important differences (to assess clinically meaningful change) that can be used for the disease-specific setting, including MS [26, 27].

While dietary intake has been implicated in the onset and clinical progression of MS, there is a paucity of studies assessing the relationship between diet and HRQoL and HSUs in pwMS. Our prior cross-sectional study found an association between higher quality-of-diet (assessed by the modified Diet Habits Questionnaire, DHQ [28]) and higher mental and physical HRQoL (MSQOL-54 [29]), such that a 1-point higher DHQ score was associated with 0.6 higher physical health scores and 0.5 higher mental health scores [30]. Another cross-sectional study by Evers and colleagues examining associations between adherence to the Dutch-Healthy Living Standard (DHLS, which recommends a high consumption of fruits/vegetables and abstaining from high salt, red meat, and processed sugar) and HRQoL and found each 1-point increase in DHLS adherence was associated with 0.41 and 0.46 higher physical and mental health scores in females, respectively [31]. We previously assessed lifestyle and clinical characteristics of the SF-6D HSU in the Health Outcomes and Lifestyle In a Sample of people with Multiple sclerosis (HOLISM) cohort at baseline, finding pwMS in the top two quartiles of total DHQ (> 80–89 and > 89–100) had 0.04 (95%CI: 0.03–0.05) and 0.05 (95%CI: 0.04–0.06) higher HSUs, respectively, than those in the bottom DHQ quartile [32]. In the Australian MS Longitudinal Study, Marck and colleagues found those in the highest vs lowest quintile of dietary quality (DHQ > 90) had 8.7% (95%CI: 1.5%–15.8%) higher AQoL-8D HSU [12]. Further, Coe and colleagues [33] found pwMS who consumed more fruits/vegetables and fiber had significantly higher EQ-5D-3L HSU, whereas higher red meat consumption showed inverse trends [34].

While there is promising evidence that quality-of-the-diet may be cross-sectionally associated with HSU, prospective relationships have not been explored. Here, we assessed short- and long-term prospective relationships of DHQ scores and HSUs over a 5-year follow-up in a large international cohort of pwMS. Based on the literature and particularly our previous cross-sectional findings, we hypothesised that higher quality-of-the-diet, as defined by a higher DHQ score [28, 35] would predict higher subsequent HSU, while meat and dairy consumption would have deleterious effects.

## Methods

### Participants and data collection

Data were extracted from the previously described HOLISM study [36, 37]. Briefly, 2,466 pwMS were recruited in 2012 via web-2.0 platforms and followed at 2.5-year intervals. Eligible participants were ≥ 18 years of age at recruitment with a self-reported clinical MS diagnosis. Ethics approval was obtained from The University of Melbourne Human Research Ethics Committee (HREC: LRR055/12).

Consenting participants completed online questionnaires related to sociodemographic, clinical, and lifestyle characteristics, as described previously [36, 37].

### SF-6D HSU

HRQoL was assessed using the SF-36 questions embedded in the MSQOL-54, from which the SF-6D HSU was estimated. The MSQOL-54 is a combination of the widely used general health QoL instrument, the SF-36, with 18 additional MS-specific questions [29]. Brazier and colleagues adapted 11 questions from these tools to develop a six-dimensional instrument, the SF-6D [38, 39], to estimate HSUs for 18,000 health states [16]. The SF-6D has been validated in multiple chronic conditions [16], including our own MS-related studies [32].

With regards to country-specific population norms, the SF-6D Australian general population norm is mean (SD) HSU 0.77 (*SD* = 0.12) [males: 0.78 (*SD* = 0.12); females 0.75 (*SD* = 0.12)]. Additionally, SF-6D HSU estimates for an Australian employed population sample were: males: mean (standard error) 0.79 (*SE* = 0.004); females 0.771 (SE = 0.003) [23]. Similarly, country-specific population norms for the United States, New Zealand, and European nations [26, 40–42] ranged between mean HSU = 0.77–0.80. The minimal important difference for the SF-6D (as a weighted mean estimate of several studies) is 0.03 (with a range of 0.01–0.05) [27].

In addition to the global HSU, the SF-6D also estimates domains for physical (physical health, pain, and vitality) and psychosocial health (role limitations, social functioning, and mental health). However, while for the total HSU score a higher score indicates better HRQoL, for the subdomains higher scores indicate worse HRQoL. Therefore, to make subdomain results more comparable to the total HSU, for these analyses HSU subdomains (range 0–4, 0–5, or 0–6) were reversed so that their measures of association were in to the same direction as total HSU, higher scores for each now indicating better HRQoL, and were rescaled to be out of 1.0.

### Quality-of-diet

Quality-of-the-diet was estimated using a modified DHQ screening tool [35, 43] comprising 24 items and ten dietary subscores: cereals, fruits/vegetables, omega-3 fatty acids, food choices, food preparation, takeaways/snacks, fat, fiber, sodium, and alcohol. Three items regarding sodium intake were removed and one item on alcohol was replaced with alternate alcohol assessment measures of frequency and volume. In addition, options for three questions in the DHQ about opting for low-fat dairy and trimming fat from meat or avoiding processed meats were modified to include “I do not consume dairy” and “I do not consume meat”. The options of nonconsumption of meat and dairy were used to define dichotomous terms used in analyses. DHQ questions are measured on a 1–5 Likert scale, with 5 signifying the ‘healthier’ self-reported dietary habits. Total DHQ score ranges between 20 and 100 with higher scores indicative of a better quality-of-the-diet and scores > 80 indicating a ‘healthier’ overall reported dietary intake.

DHQ scores and subscores were evaluated as both continuous and quartile categorical terms. Continuous total DHQ score was rescaled as a 10-unit term for more interpretable coefficients, but other terms were evaluated as-is.

### Other covariates

All measures were queried at each review unless otherwise specified. Age was derived from self-reported birthdate at baseline. Sex was queried at baseline as male/female. Highest completed education was queried at baseline (primary/secondary/graduate/postgraduate). Physical activity was assessed using the International Physical Activity Questionnaire (IPAQ), defining levels as inactive, minimally active, and active as per IPAQ guidelines [43]. BMI was calculated from self-reported height (metres) and weight (kilograms) by the function weight/height^2^. Smoking and alcohol intake behaviours were queried, smoking categorised as never/ex-smoker/current smoker, and alcohol categorised as none/moderate/heavy as per WHO increments [44]. Vitamin D and omega-3 supplement use were also queried.

Disability was self-reported using the Patient Determined Disease Steps (PDDS) from which duration-adjusted Patient-derived Multiple Sclerosis Severity Score (P-MSSS) was derived [45, 46]. Fatigue was measured by the Fatigue Severity Scale (FSS), mean FSS > 5 indicating clinically significant fatigue [47]. Depression was assessed by the Patient Health Questionnaire-2 (PHQ-2), total PHQ-2 > 2 indicating depression-risk [48]. The Self-administered Comorbidity Questionnaire (SCQ) [49] queried a list of comorbidities at baseline. Additionally, participants reported if they were taking prescription antidepressant and/or anti-fatigue medications, and whether they were experiencing ongoing symptoms due to recent relapse in the preceding 30 days.

### Statistical analysis

Cross-sectional relationships of DHQ with HSUs were assessed by linear regression. The prospective associations of DHQ with subsequent HSUs were assessed via panel-data analyses using generalised estimating equations (GEE) [50] for lagged exposure variables, exploring the association between diet at baseline with 2.5-year HSU and diet at 2.5-year with 5-year HSU. These longitudinal analyses using GEEs estimate a marginal model, providing interpretable estimates for relationships while allowing for correlated observations within individuals. To assess the short and long-term relationships of DHQ with subsequent HSU, baseline DHQ was evaluated for its relationships with HSU at the 2.5-year and 5-year follow-up via linear regression. In addition, the relationships of DHQ with SF-6D subdomains were evaluated by quantile regression, estimating differences in medians.

Multivariable models were developed based on a priori reasoning and those factors which were independently associated with quality-of-the-diet and HSU, and then assessed in directed acyclic graphs. In addition, factors materially differing in bias analyses comparing characteristics between those included and not included in analyses were evaluated for potential inclusion in multivariable models. All cross-sectional and prospective DHQ-HSU analyses were adjusted for whether participants were experiencing ongoing symptoms due to a recent relapse at each review. Prospective models were also adjusted for baseline HSU or HSU subdomain, as appropriate. Full multivariable analyses further controlled for age, sex, education, disability, clinically significant fatigue, prescription antidepressant medication use, treated comorbidity number, and depression-risk (PHQ-2).

Sensitivity analyses adjusting HSU models for BMI and physical activity were also undertaken. However, BMI and physical activity were not included in the primary multivariable models due to being partially/completely on the causal pathway between diet and HSU.

All statistical analyses were conducted in STATA/SE 15.0 (StataCorp, College Station, USA).

All analyses were complete case.

## Results

### Cohort characteristics

The analysis sample included 839 participants who provided data at all three timepoints – baseline, and 2.5- and 5-year follow-up reviews (Supplemental Fig. 1). The cohort was predominately female (82.4%), of mean age 50.9 years, with the representative countries including Australia (34.4%), United States (22.6%), United Kingdom (16.3%), and New Zealand (11.4%), among others. The mean HSU for the study cohort, 0.71, lower than the general population norms of 0.77–0.80 utility points for comparable country-specific population norms [26, 40–42]. Over two-thirds (69.9%) were of RRMS phenotype, 32.2% had moderate or severe disability, 38.6% had clinically significant fatigue, and roughly 10% screened positive for depression-risk. The cohort generally engaged in healthy behaviours: 55.8% had never smoked, around 65% were at least minimally active, and 65.2% had underweight/normal BMI. The mean DHQ was 82.9/100, and 54.1% reported consuming meat and 50.6% dairy.

**Fig. 1:**
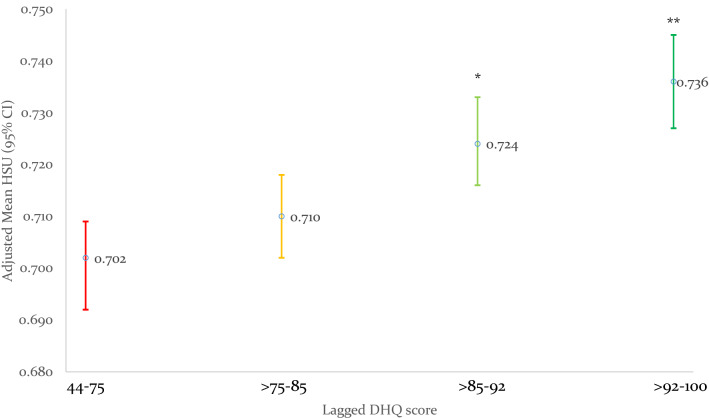
Prospective relationships of pooled lagged total Diet Habits Questionnaire (DHQ) score quartile with subsequent HSU 2.5 years later

Compared to those lost to attrition, participants in the analysis sample were more likely to have completed a tertiary qualification (68.2%), be at least minimally active (65.0%), and take vitamin D supplements (85.3%, Supplemental Table 1). Additionally, fewer were depressed (10.2%), severely disabled (10.7%), had clinically significant fatigue (38.6%), suffered from three or more comorbidities (11.2%), were obese (13.9%), or were current smokers (7.4%).

**Table 1:** Prospective pooled lagged panel-data associations between DHQ and subsequent change in HSU

Category	aβ (95%CI)^a^	aβ (95%CI)^b^
DHQ total score, 10-unit continuous	**0.012 (0.007, 0.016)**	**0.010 (0.005, 0.014)**
	***p* < *0.001***	***p* < *0.001***
< 75	0.00 [Ref]	0.00 [Ref]
> 75–85	0.01 (− 0.00, 0.03)	0.01 (− 0.00, 0.02)
> 85–92	**0.03 (0.01, 0.04)**	**0.02 (0.01, 0.04)**
> 92–100	**0.03 (0.02, 0.05)**	**0.03 (0.01, 0.04)**
*Trend*	***p* < *0.001***	***p* < *0.001***
DHQ fat subscore, continuous	**0.020 (0.011, 0.030)**	**0.016 (0.007, 0.025)**
	***p* < *0.001***	***p* < *0.001***
< 3.7	0.00 [Ref]	0.00 [Ref]
> 3.7–4.2	0.01 (− 0.00, 0.02)	0.01 (− 0.00, 0.02)
> 4.2–4.7	**0.02 (0.01, 0.04)**	**0.01 (0.00, 0.03)**
> 4.7–5	**0.03 (0.02, 0.05)**	**0.02 (0.01, 0.04)**
*Trend*	***p* < *0.001***	***p* = *0.001***
DHQ cereal subscore, continuous	**0.008 (0.003, 0.014)**	**0.007 (0.002, 0.013)**
	***p* = *0.003***	***p* = *0.009***
< 3	0.00 [Ref]	0.00 [Ref]
> 3–4	**0.01 (0.00, 0.03)**	**0.01 (0.00, 0.03)**
> 4–4.3	**0.02 (0.00, 0.03)**	**0.02 (0.00, 0.03)**
> 4.3–5	**0.02 (0.01, 0.04)**	**0.02 (0.01, 0.04)**
*Trend*	***p* = *0.001***	***p* = *0.004***
DHQ fruit and vegetable subscore, continuous	**0.011 (0.004, 0.018)**	**0.010 (0.003, 0.016)**
	***p* = *0.002***	***p* = *0.004***
< 3.2	0.00 [Ref]	0.00 [Ref]
> 3.2–4	0.00 (0.01, 0.02)	0.00 (− 0.01, 0.01)
> 4–4.4	**0.02 (0.01, 0.04)**	**0.02 (0.01, 0.04)**
> 4.4–5	**0.02 (0.01, 0.04)**	**0.02 (0.00, 0.03)**
*Trend*	***p* = *0.001***	***p* = *0.001***
DHQ takeaway subscore, continuous	**0.012 (0.005, 0.019)**	**0.010 (0.003, 0.016)**
	***p* < *0.001***	***p* = *0.004***
< 3.7	0.00 [Ref]	0.00 [Ref]
> 3.7–4.3	0.01 (0.00, 0.02)	0.01 (− 0.01, 0.02)
> 4.3–5	**0.02 (0.01, 0.03)**	**0.02 (0.00, 0.03)**
*Trend*	***p* = *0.005***	***p* = *0.014***
DHQ food choices subscore, continuous	**0.011 (0.005, 0.018)**	**0.009 (0.002, 0.015)**
	***p* = *0.001***	***p* = *0.006***
< 3.8	0.00 [Ref]	0.00 [Ref]
> 3.8–4.5	0.01 (0.01, 0.02)	0.00 (-0.01, 0.02)
> 4.5–5	**0.07 (0.01, 0.04)**	**0.02 (0.01, 0.03)**
*Trend*	***p* < *0.001***	***p* = *0.001***
DHQ omega-3 subscore, continuous	**0.005 (0.001, 0.009)**	0.004 (− 0.000, 0.007)
	***p* = *0.014***	*p* = *0.070*
< 3	0.00 [Ref]	0.00 [Ref]
> 3–4	**0.02 (0.00, 0.03)**	0.01 (− 0.00, 0.02)
> 4–5	**0.02 (0.01, 0.03)**	**0.02 (0.01, 0.03)**
*Trend*	***p* = *0.001***	***p* = *0.005***
DHQ food preparation subscore, continuous	**0.014 (0.005, 0.023)**	**0.011 (0.002, 0.019)**
	***p* = *0.002***	***p* = *0.015***
< 4.4	0.00 [Ref]	0.00 [Ref]
> 4.4–5	0.01 (0.00, 0.02)	0.01 (− 0.00, 0.02)
	*p* = *0.07*	*p* = *0.15*
DHQ fiber subscore, continuous	**0.014 (0.006, 0.022)**	**0.012 (0.005, 0.020)**
	***p* < *0.001***	***p* = *0.001***
< 3.3	0.00 [Ref]	0.00 [Ref]
> 3.3–3.9	**0.02 (0.00, 0.03)**	**0.02 (0.01, 0.03)**
> 3.9–4.4	**0.02 (0.01, 0.04)**	**0.02 (0.01, 0.03)**
> 4.4–5	**0.03 (0.01, 0.04)**	**0.03 (0.01, 0.04)**
*Trend*	***p* = *0.001***	***p* = *0.002***
Consumes meat		
No	0.00 [Ref]	0.00 [Ref]
Yes	**− 0.03 (− 0.04, -0.02)**	**− 0.02 (− 0.03, -0.01)**
	***p* < *0.001***	***p* = *0.001***
Consumes dairy		
No	0.00 [Ref]	0.00 [Ref]
Yes	**− 0.02 (− 0.03, -0.00)**	**− 0.01 (− 0.02, − 0.00)**
	***p* = *0.004***	***p* = *0.008***

Analysis by lagged panel-data linear regression, estimating *β* [95% confidence intervals (CI)]

Boldface denotes significance (*p* < 0.05)

^a^Model 1 adjusted for experiencing ongoing symptoms due to relapse and baseline HSU

^b^Model 2 further adjusted for age, sex, level of highest education, disability (P-MSSS), clinically significant fatigued, prescription antidepressant medication use, treated comorbidity number, and depression-risk (PHQ-2)

*aβ* Adjusted beta coefficient; *DHQ* Dietary Habits Questionnaire; *HSU* Health State Utility

### Cross-sectional DHQ characteristics of HSU at 2.5- and 5-year reviews

In cross-sectional analyses, dose-dependent positive associations between total DHQ score and HSU were found at both the 2.5- and 5-year reviews (Supplemental Table 2). Each 10-unit increase in total DHQ was associated with 0.012 and 0.009 higher HSU at 2.5- and 5-year reviews, respectively, and those in the top quartile of DHQ had 0.06 (95% CI = 0.04–0.08) and 0.04 (95% CI = 0.01–0.07) higher HSU at each review. Among the DHQ subscores, fat, fiber, and cereal showed the strongest and most consistent associations. Meat and dairy consumption were each associated with 0.02 lower HSU at 2.5 and 5-year reviews, respectively.

**Table 2:** Prospective DHQ characteristics of HSU at 2.5 and 5-year

	2.5-year			5-year		
	*n* (%)	*aβ* (95%CI)^a^	*aβ* (95%CI)^b^	*n* (%)	*aβ* (95%CI)^a^	*aβ* (95%CI)^b^
DHQ total score, 10-unit continuous		**0.028 (0.020, 0.036)**	**0.012 (0.005, 0.020)**		**0.025 (0.018, 0.033)**	**0.008 (0.001, 0.015)**
		***p* < *0.001***	***p* = *0.001***		***p* < *0.001***	***p* = *0.021***
< 75	177 (25.8%)	0.00 [Reference]	0.00 [Reference]	186 (25.6%)	0.00 [Reference]	0.00 [Reference]
> 75–85	185 (27.0%)	**0.02 (0.00, 0.04)**	0.01 (− 0.01, 0.03)	201 (27.6%)	**0.02 (0.00, 0.04)**	0.01 (− 0.01, 0.03)
> 85–92	166 (24.2%)	**0.03 (0.01, 0.05)**	0.02 (− 0.00, 0.04)	169 (23.2%)	**0.02 (0.00, 0.04)**	0.01 (− 0.01, 0.03)
> 92–100	158 (23.0%)	**0.04 (0.02, 0.06)**	**0.03 (0.01, 0.05)**	172 (23.6%)	**0.04 (0.02, 0.06)**	**0.03 (0.00, 0.05)**
*Trend*		***p* < *0.001***	***p* = *0.007***		***p* < *0.001***	***p* = *0.031***
DHQ fat subscore, continuous		**0.050 (0.034, 0.065)**	**0.022 (0.008, 0.035)**		**0.045 (0.030, 0.061)**	**0.015 (0.001, 0.029)**
		***p* < *0.001***	***p* = *0.002***		***p* < *0.001***	***p* = *0.033***
< 3.7	192 (28.0%)	0.00 [Reference]	0.00 [Reference]	204 (28.0%)	0.00 [Reference]	0.00 [Reference]
> 3.7–4.2	178 (26.0%)	**0.03 (0.01, 0.05)**	**0.03 (0.01, 0.04)**	189 (26.0%)	0.02 (− 0.00, 0.04)	0.01 (− 0.01, 0.03)
> 4.2–4.7	149 (21.7%)	**0.02 (0.00, 0.04)**	0.01 (− 0.01, 0.03)	156 (21.4%)	0.01 (− 0.01, 0.03)	− 0.00 (− 0.02, 0.02)
> 4.7–5	167 (24.3%)	**0.04 (0.02, 0.06)**	**0.03 (0.01, 0.05)**	179 (24.6%)	**0.04 (0.02, 0.06)**	**0.02 (0.00, 0.04)**
*Trend*		***p* = *0.001***	***p* = *0.019***		***p* = *0.001***	*p* = *0.075*
DHQ cereal subscore, continuous		**0.022 (0.013, 0.032)**	**0.009 (0.000, 0.017)**		**0.020 (0.011, 0.030)**	0.007 (− 0.001, 0.016)
		***p* < *0.001***	***p* = *0.045***		***p* < *0.001***	*p* = *0.10*
< 3	176 (25.7%)	0.00 [Reference]	0.00 [Reference]	182 (25.0%)	0.00 [Reference]	0.00 [Reference]
> 3–4	228 (33.3%)	**0.02 (0.01, 0.04)**	**0.02 (0.00, 0.04)**	246 (33.8%)	**0.02 (0.00, 0.04)**	0.01 (− 0.00, 0.03)
> 4–4.3	111 (16.2%)	0.02 (− 0.00, 0.04)	0.01 (− 0.01, 0.04)	120 (16.5%)	0.02 (− 0.01, 0.04)	0.01 (− 0.01, 0.03)
> 4.3–5	170 (24.8%)	**0.03 (0.01, 0.05)**	**0.02 (0.00, 0.04)**	179 (24.6%)	**0.03 (0.01, 0.05)**	0.02 (− 0.00, 0.04)
*Trend*		***p* = *0.013***	*p* = *0.060*		***p* = *0.013***	*p* = *0.10*
DHQ fruit/vegetable subscore, continuous		**0.031 (0.020, 0.042)**	**0.015 (0.005, 0.024)**		**0.027 (0.017, 0.038)**	0.008 (− 0.002, 0.018)
		***p* < *0.001***	***p* = *0.003***		***p* < *0.001***	*p* = *0.11*
< 3.2	178 (26.05)	0.00 [Reference]	0.00 [Reference]	191 (26.2%)	0.00 [Reference]	0.00 [Reference]
> 3.2–4	206 (30.05)	0.01 (− 0.01, 0.03)	0.01 (− 0.01, 0.03)	215 (29.5%)	0.02 (− 0.00, 0.04)	0.01 (− 0.01, 0.03)
> 4–4.4	144 (21.0%)	0.02 (− 0.00, 0.04)	0.01 (− 0.01, 0.03)	154 (21.2%)	0.02 (− 0.00, 0.04)	0.01 (− 0.01, 0.03)
> 4.4–5	158 (23.0%)	**0.03 (0.01, 0.05)**	**0.03 (0.01, 0.05)**	168 (23.1%)	**0.03 (0.00, 0.05)**	0.02 (− 0.00, 0.04)
*Trend*		***p* = *0.009***	***p* = *0.018***		***p* = *0.020***	*p* = *0.15*
DHQ takeaway subscore, continuous		**0.027 (0.016, 0.038)**	**0.012 (0.002, 0.022)**		**0.026 (0.015, 0.037)**	**0.010 (0.000, 0.019)**
		***p* < *0.001***	***p* = *0.015***		***p* < *0.001***	***p* = *0.036***
< 3.7	192 (28.9%)	0.00 [Reference]	0.00 [Reference]	200 (28.4%)	0.00 [Reference]	0.00 [Reference]
> 3.7–4.3	165 (24.8%)	0.01 (− 0.01, 0.02)	− 0.00 (− 0.02, 0.02)	176 (25.0%)	0.02 (− 0.00, 0.04)	0.01 (− 0.01, 0.03)
> 4.3–5	308 (46.3%)	0.01 (− 0.00, 0.03)	0.01 (− 0.01, 0.03)	329 (46.7%)	**0.02 (0.00, 0.04)**	0.01 (− 0.00, 0.03)
*Trend*		*p* = *0.14*	*p* = *0.29*		***p* = *0.019***	*p* = *0.14*
DHQ food choices subscore, continuous		**0.031 (0.020, 0.042)**	**0.014 (0.004, 0.024)**		**0.029 (0.18, 0.040)**	**0.011 (0.002, 0.021)**
		***p* < *0.001***	***p* = *0.005***		***p* < *0.001***	***p* = *0.020***
< 3.8	180 (26.6%)	0.00 [Reference]	0.00 [Reference]	188 (26.2%)	0.00 [Reference]	0.00 [Reference]
> 3.8–4.5	184 (27.1%)	0.02 (− 0.00, 0.04)	0.01 (− 0.01, 0.03)	194 (27.0%)	0.02 (− 0.00, 0.04)	0.01 (− 0.01, 0.03)
> 4.5–5	314 (46.3%)	**0.03 (0.02, 0.05)**	**0.02 (0.01, 0.04)**	337 (46.9%)	**0.03 (0.01,0.05)**	0.02 (− 0.00, 0.04)
*Trend*		***p* < *0.001***	***p* = *0.009***		***p* = *0.001***	*p* = *0.067*
DHQ omega -3 subscore, continuous		**0.012 (0.006, 0.019)**	0.004 (− 0.002, 0.010)		**0.014 (0.007, 0.021)**	**0.006 (0.000, 0.012)**
		***p* < *0.001***	*p* = *0.15*		***p* < *0.001***	***p* = *0.040***
< 3	260 (37.9%)	0.00 [Reference]	0.00 [Reference]	274 (37.6%)	0.00 [Reference]	0.00 [Reference]
> 3–4	151 (22.0%)	− 0.00 (− 0.02, 0.02)	− 0.01 (− 0.02, 0.01)	165 (22.7%)	− 0.00 (− 0.02, 0.02)	− 0.01 (− 0.03, 0.01)
> 4–5	275 (40.1%)	**0.02 (0.00, 0.04)**	**0.02 (0.00, 0.03)**	289 (39.7%)	**0.02 (0.01, 0.04)**	0.02 (− 0.00, 0.03)
*Trend*		***p* = *0.010***	***p* = *0.035***		***p* = *0.004***	*p* = *0.062*
DHQ food preparation subscore, continuous		**0.033 (0.016, 0.050)**	0.013 (− 0.001, 0.027)		**0.028 (0.011, 0.044)**	0.006 (− 0.009, 0.020)
		***p* < *0.001***	*p* = *0.071*		***p* = *0.001***	*p* = *0.44*
< 4.4	178 (26.5%)	0.00 [Reference]	0.00 [Reference]	185 (26.05)	0.00 [Reference]	0.00 [Reference]
> 4.4–5	495 (73.6%)	**0.02 (0.00, 0.03)**	0.01 (− 0.00, 0.03)	528 (74.1%)	0.02 (− 0.00, 0.03)	0.01 (− 0.01, 0.03)
		***p* = *0.038***	*p* = *0.13*		*p* = *0.070*	*p* = *0.29*
DHQ fiber subscore, continuous		**0.038 (0.026, 0.051)**	**0.018 (0.007, 0.029)**		**0.034 (0.022, 0.047)**	**0.011 (0.000, 0.022)**
		***p* < *0.001***	***p* = *0.002***		***p* < *0.001***	***p* = *0.042***
< 3.3	166 (24.9%)	0.00 [Reference]	0.00 [Reference]	172 (24.4%)	0.00 [Reference]	0.00 [Reference]
> 3.3–3.9	174 (26.1%)	0.02 (− 0.01, 0.04)	**0.02 (0.00, 0.04)**	190 (27.0%)	0.01 (− 0.01, 0.03)	0.01 (− 0.01, 0.03)
> 3.9–4.4	185 (27.85)	**0.03 (0.01,0.05)**	**0.02 (0.00, 0.04)**	194 (27.5%)	0.02 (− 0.00, 0.04)	0.01 (− 0.02, 0.03)
> 4.4–5	141 (21.2%)	**0.03 (0.01, 0.05)**	**0.03 (0.01, 0.05)**	149 (21.1%)	**0.03 (0.00, 0.05)**	0.02 (− 0.01, 0.04)
*Trend*		***p* = *0.005***	***p* = *0.012***		***p* = *0.019***	*p* = *0.17*
Consumes meat						
No	315 (45.9%)	0.00 [Reference]	0.00 [Reference]	337 (46.3%)	0.00 [Reference]	0.00 [Reference]
Yes	371 (54.1%)	**− 0.02 (− 0.04, − 0.01)**	− 0.01 (− 0.03, 0.00)	391 (53.7%)	**− 0.02 (− 0.04, − 0.01)**	− 0.01 (− 0.03, 0.00)
		***p* = *0.002***	*p* = *0.069*		***p* = *0.001***	*p* = *0.11*
Consumes dairy						
No	334 (49.1%)	0.00 [Reference]	0.00 [Reference]	354 (49.0%)	0.00 [Reference]	0.00 [Reference]
Yes	346 (50.9%)	**− 0.02 (− 0.04, − 0.01)**	**− 0.02 (− 0.03, − 0.01)**	368 (51.0%)	**− 0.03 (− 0.04, − 0.01)**	**− 0.02 (− 0.03, − 0.01)**
		***p* = *0.001***	***p* = *0.006***		***p* < *0.001***	***p* = *0.008***

Boldface denotes significance (p < 0.05). Analysis by linear regression, estimating β (95% confidence intervals (CI))

^a^Model 1 adjusted for experiencing ongoing symptoms due to relapse at each timepoint and baseline HSU

^b^Model 2 further adjusted for age, sex, level of highest education, disability (P-MSSS), clinically significant fatigue, prescription antidepressant medication use, treated comorbidity number, and depression-risk (PHQ-2)

*aβ* adjusted Beta coefficient; *DHQ* Diet Habits Questionnaire

### Prospective analysis of HSU at 2.5- and 5-year reviews

In pooled analyses, each 10-unit increase in total DHQ score was associated with 0.012 higher HSU. Total DHQ also showed a robust dose-dependent positive association with HSU, such that those in the top two quartiles of total DHQ had 0.03 higher HSU, persisting on adjustment (Table 1, Fig. 1). Of the DHQ subscores, all but food preparation showed strong and robust associations with higher HSU. The strongest associations were seen for the fiber subscore. Meat and dairy consumption were each associated with 0.02 and 0.01 lower HSU, respectively. These pooled results were broadly supported by evaluating each 2.5-year interval, with baseline DHQ measures associated with 2.5-year HSU and 2.5-year DHQ measures with 5-year HSUs. (Supplemental Table 3).

Evaluating HSU subdomains, the positive association of total DHQ and total HSU was most evident for the pain subdomain, with each 10-unit increase in total DHQ score associated with 0.022 higher median pain score and those in the top quartile of DHQ score having 0.08 higher median pain score. The physical health and vitality subdomains also showed strong associations (Supplemental Table 4). The strongest negative associations of meat or dairy DHQ subscores were seen for the pain HSU subdomain, each associated with 0.05 lower median pain HSU, with negative relationships also seen for meat consumption and physical health (-0.03) and vitality (-0.02), and for dairy consumption and vitality HSU subdomains (-0.02).

### Short- and long-term prospective associations of baseline diet parameters and subsequent HSU at 2.5 and 5-year reviews

**Fig. 2:**
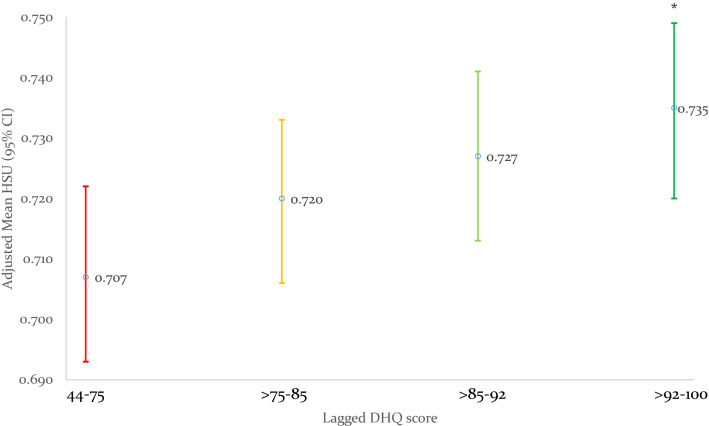
Association of baseline total Diet Habits Questionnaire (DHQ) score quartile with Health State Utilities (HSU) at 2.5-years

**Fig. 3:**
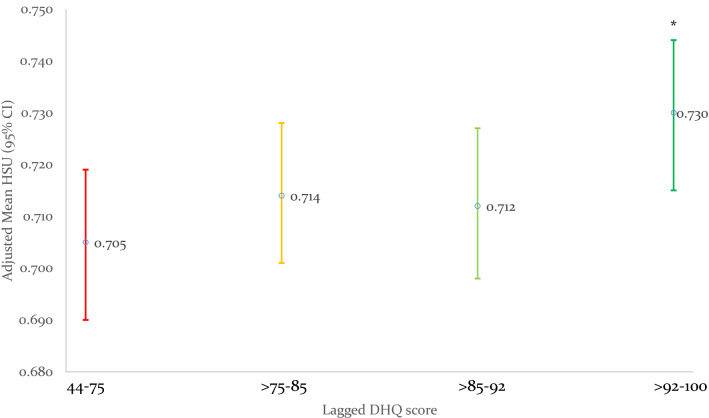
Distribution of baseline total Diet Habits Questionnaire (DHQ) score quartile by Health State Utilities (HSU) at 5-years

Baseline total DHQ score was associated with improved HSU at both 2.5 and 5-year reviews, with each 10-unit increase in baseline DHQ associated with 0.012 and 0.008 higher HSU at each review, and with those in the top DHQ quartile having 0.03 higher HSU at 2.5 and 5-year reviews, respectively (Table 2, Figs. 2 and 3). Baseline fat consumption showed the strongest adjusted association with HSU at the 2.5- and 5-year review, while fiber, and to a lesser extent fruit/vegetable DHQ subscores, were associated with higher HSU at the 2.5-year review but not at the 5-year review. Baseline dairy consumption was consistently associated with 0.02 lower HSU at 2.5- and 5-year reviews, but baseline meat consumption was not significantly associated with HSU at either timepoint.

Positive associations of baseline DHQ scores with total HSU were most evident for the pain, physical health, and vitality HSU subdomains (Supplemental Table 5). Among DHQ subscores, fat and fruit/vegetable were most strongly positively associated, with fat showing strong and consistent associations with pain, while fruit/vegetable was strongly associated with vitality. Meat consumption, while not associated with total HSU, was significantly associated with lower physical health, whereas dairy consumption was associated with lower overall HSU but not with any individual HSU subdomains.

### Sensitivity analyses

While sensitivity analysis covariates were not included in our main multivariable models, there was a justification to evaluate further adjustment for BMI and physical activity on the associations of quality-of-the-diet and HSU. In all prospective analyses, further adjustment for BMI and physical activity had no impact on the diet-HSU associations (data not shown).

## Discussion

Using HSUs as a measure of HRQoL, we found that better quality-of-diet at baseline was predictive of higher HSU up to 5-years later, with higher baseline DHQ scores associated with 0.02–0.03 higher HSU at 2.5- and 5-year reviews, respectively. These results are at or close to the threshold for a minimally clinically important difference of 0.03 [27], indicating the potential HRQoL benefit of quality-of-the-diet for pwMS [27]. Of the DHQ subscores, the strongest associations with total HSU were seen for fat and fiber subscores, evident cross-sectionally and in prospective analyses. Further, dairy consumption was associated with 0.02 lower HSUs, though meat consumption was not robustly associated. Of the HSU subdomains, the strongest and most consistent prospective associations were seen for the pain, physical health, and vitality subdomains.

HSUs are an understudied aspect in epidemiological studies in MS, despite being a critical nexus point between the assessment of HRQoL in this population and the translation of research findings to practice and policy. Nonetheless, there is a dearth of literature on the relationship of diet and HSUs in pwMS and these were previously only cross-sectional in nature [31, 52]. Our study addresses this gap, with HSU results potentially of use in health economics MS-prevention models. We investigated the prospective relationships of diet and HSU via two different analyses, enabling both overall, as well as short and long-term effects, to be assessed. In the main prospective analysis evaluating pooled results at each 2.5-year interval, we observed a dose-dependent positive association of total DHQ score with HSU outcomes at both the 2.5- and 5-year review. Evaluated as a continuous 10-unit term, total DHQ score was associated with 0.010 higher HSU, and each higher quartile of total DHQ was associated with 0.01 higher HSU, and the highest quartile (> 92–100) of total DHQ had 0.03 higher HSU than the reference (< 72), in line with the minimum clinically important difference for the SF-6D. We also evaluated short- and long-term effects, showing that the associations of baseline DHQ with subsequent HSU 2.5 and 5-years later were comparable, each 10-unit increase in continuous DHQ associated with 0.012 and 0.008 higher HSU, and those in the top quartile having 0.03 higher HSU. This substantiates the pooled prospective analyses we present, while also suggesting that improved dietary quality may have short and long-term beneficial impacts on HSU in this population. It would be of interest to evaluate these dynamics further in a population surveyed at more frequent intervals, to ascertain how quickly diet may realise such positive effects. Subject to such replication, interventional studies of quality-of-the-diet improvement could then be implemented with a goal to validating the utility of diet in improving HSUs and HRQoL.

In general, our results here are in keeping with previous studies, including our own cross-sectional analysis of HOLISM data at baseline [32]. The cross-sectional results of the present study show a dose-dependent positive association at both 2.5- and 5-year reviews, similar in magnitude to that previously observed [32]. Marck and colleagues also showed cross-sectional relationships between quality-of-the-diet and HSU [12] in the Australian MS Longitudinal Study (AMSLS), with participants in the top two quartiles (80–90 and > 90) of DHQ scores associated with 6.6 and 8.7% higher HSU as measured by the AQoL-8D (out of 100%), respectively, with dose–response patterns. While the AMSLS analyses found no associations between meat or dairy consumption and HSUs [51], both the present study and our baseline study of the HOLISM cohort showed consistent and robust negative associations [30]. The AMSLS cohort had a slightly lower overall dietary quality (mean DHQ = 73.4) than the HOLISM study (mean DHQ = 82.9), and lower proportions of pwMS who avoided meat and dairy (7.9% and 8.1%, respectively) compared with HOLISM (baseline [32]: 27% and 38%, respectively). Marck and colleagues utilised the AQoL-8D, which is known to be preferentially sensitive to psychosocial health, whereas previous studies have noted that the SF-6D is preferentially sensitive to physical HRQoL [17, 53]. Relationships between diet and HSUs have also been explored in other studies with distinct methods. Coe and colleagues cross-sectionally evaluated individual food and nutritional components in the UK MS Register cohort, and found higher fish intake and nutrient content were associated with better EQ-5D-3L, and meat consumption associated with worse fatigue and anxiety/depression dimensions of the EQ-5D, and increased pain [33]. Despite the differences in diet and HSU measures, all studies had similar cross-sectional results for overall dietary quality and HSU, with this external consistency supportive of our prospective results.

Of the HSU subdomains, the strongest and most consistent prospective associations were seen for the pain subdomain, with those in the top quartile of total DHQ having 0.08 higher score in the lagged analysis and 0.05–0.08 higher scores in the prospective analyses at 2.5- and 5-year reviews. Positive associations were also seen for the physical health and vitality subdomains. The associations of better quality-of-the-diet with physical health and vitality are somewhat intuitive and align with the expectation of improved diet acting upon perceived physical health. The mechanisms underlying the associations seen for the pain subdomain, however, are less apparent. The pain HSU subdomain comprises two questions regarding how much pain a participant has and how much this interferes with activities and quality of life. Pain could act alongside or via disability, fatigue, and/or depression but our models controlled for these factors. Also, adjustment for prescription analgesic medication did not affect our results (data not shown). Studies of diet and HSU, particularly using the SF-6D, represent an understudied area, and comparators for the relationships observed are sparse. An RCT of a multimodal lifestyle intervention that included diet in a population at risk of diabetes assessed HSU using the SF-6D [53], finding that those in the intervention group had a 1.6-point higher pain subdomain score, though this association was abrogated on adjustment for change in BMI. We found in our sensitivity analyses that the diet-HSU relationships were independent of BMI, but this could reflect differences in the population or the RCT design of the Florez study. The mechanisms by which diet could impact upon pain-related HRQoL merit further study.

### Potential mechanisms

In addition to total DHQ score, the strongest associations among the DHQ subscores were seen for fat and fiber. Studies have found that elevated consumption of saturated and trans-fats are associated with promotion of neuroinflammatory agents, which may explain previously reported associations between unhealthy fats and increased frequencies of fatigue, disability, and depression in pwMS [11, 12, 30]. However, the DHQ fat score does not distinguish between healthy (unsaturated) and unhealthy fat (saturated) consumption, but only indicates overall fat-related dietary behaviours [28]. Thus, further research exploring the role of different types of fat/s in HSU is advised. Similarly, associations with fiber may be explained via actions on the gut microbiome as studies in both human and animal models have shown impacts of diet change [55, 56].

It is also possible that the observed relationships of DHQ and HSU found in the present study reflect some degree of confounding or action by other related pathways. While we adjusted for confounding by demographic and clinical characteristics, particularly disability, fatigue, and depression, it is possible that some residual confounding may explain these results. Another possibility lies in the covariation of quality-of-the-diet with other lifestyle behaviours and related factors, including physical activity and BMI. We did not control for these characteristics in our main multivariable models as these characteristics also lie in some part on the potential causal pathway between diet and HSU. In sensitivity analyses, however, we found that the prospective diet-HSU relationships were robust to adjustment for BMI and physical activity. However, given the covariation of diet with these parameters it should be examined in other samples to determine what extent diet acts outside these mechanisms.

### Strengths and limitations

Despite appreciable attrition, the large cohort assessed in the present study allows good statistical power to assess dietary relationships. The HOLISM study captured a range of sociodemographic, clinical, and lifestyle data, allowing the ability to study multiple predictor variables and evaluation of multivariable adjustment models controlling for possible confounders. This enables confidence in the observed associations as being indicative of a DHQ-HSU relationship. Cohort demographic and clinical characteristics are typical of other MS cohorts [6, 52], suggesting that this cohort is broadly representative of the MS community, thereby contributing to the generalisability of results. That said, the cohort had a preponderance of healthy lifestyle behaviours, particularly the reported diet, where only 25% of the cohort had a diet score < 75 out of 100, which may limit the generalisability of results. In addition, the mean HSU of the retained cohort was 0.71, which is below the SF-6D Australian general population norm of 0.78 [23]. This being so, there may be some issues of generalisability to less healthy populations, particularly those of higher disability or persons with progressive MS phenotypes. Studies of diet-HSU relationships in such populations should be undertaken with a goal to assess whether these associations differ by clinical severity. Finally, the study is susceptible to participation and reporting biases, as there is a notable healthy participation bias both in initial participation and subsequent retention, and there is potential for reporting biases in the diet parameters and other factors queried.

The 20-item DHQ is also limited compared with other comprehensive measures of quality-of-the-diet, such as the EPIC-Norfolk FFQ used by Coe and colleagues [33]. The DHQ provides a general snapshot into dietary quality in the form of a single score as opposed to specific measures for each element of dietary habits but lacks items concerning nutrient or energy levels, precluding cross-referencing data to confirm whether participants have over- or under-reported their dietary consumption. However, the DHQ remains an effective form of dietary measurement, compared with the comprehensive FFQs utilised in other studies [12, 30], and its brevity allows for greater completion than other instruments [56], whilst allowing for a number of confounders to be explored. As such, the DHQ was deemed appropriate and while it may have a disposition for measurement error, this can be controlled for [35]. However, a more comprehensive measure of diet is advised in future studies examining diet-HSU relationships, both to substantiate our results and to determine which aspects of diet account for these relationships.

## Conclusions

This longitudinal observational study supports a strong and robust prospective association between a higher quality diet and higher HSU in pwMS. Those who maintained the healthiest dietary practices as defined by the DHQ had significantly higher HSU up to 5 years later. These results support the findings in our previous baseline study [32], and reinforce the relationship between the quality-of-the-diet and HRQoL in pwMS. Higher scores in the fat and fiber DHQ subscores appear to be important dietary factors for higher HSU. Subject to replication and substantiation in clinical trials, behaviour modification interventions to improve the quality-of-the-diet of participants' diet may be a potential point of intervention to improve health and wellbeing in pwMS.

## Supplementary Information

Below is the link to the electronic supplementary material.

Supplementary file1 (DOCX 285 KB)

## Data Availability

Persons interested in acquiring de-identified data extracts for the data underlying these analyses may contact the corresponding author.
