# Supplementary material for: Higher diet quality is associated with short and long-term benefits on SF-6D health state utilities: a 5-year cohort study in an international sample of people with multiple sclerosis
Source: Qual Life Res. 2023 Feb 23;32(7):1883–96. doi: 10.1007/s11136-023-03361-w (PMC10241689; doi:10.1007/s11136-023-03361-w)
Supplement: Supplementary file 1 — Supplementary file1 (DOCX 285 KB) [file 11136_2023_3361_MOESM1_ESM.docx]

*Supplemental Figure 1. Flowchart of participants for whom an HSU could be generated for within the HOLISM study from baseline to 5-year review*


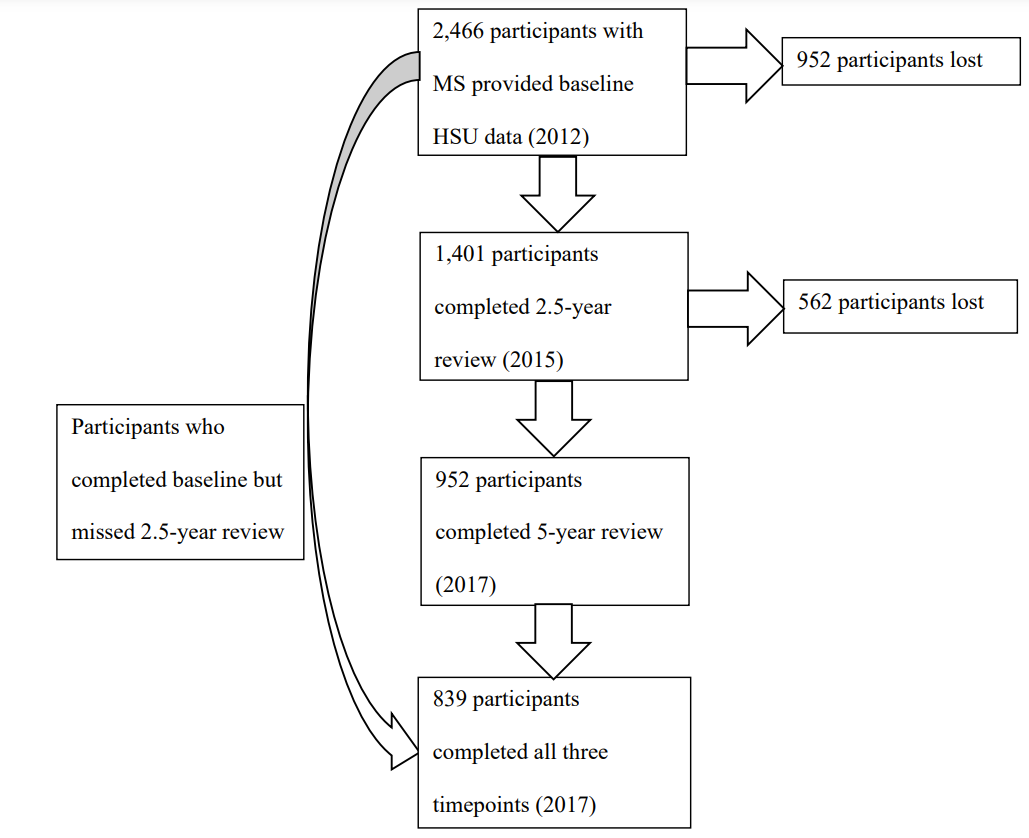


*Supplemental Table 1: Baseline characteristics of the analysis sample and comparison with the remainder of the cohort not included.*

|  | Not retained at 2.5-year and 5-year follow-up from baseline | Retained at all time points | *Difference* |
| --- | --- | --- | --- |
|  | Number of participants (%) | |  |
| Sex  Male  Female  (Missing) | 269 (17.5%) 1266 (82.5%) (92 (5.7%)) | 148 (17.6%) 691 (82.4%) (0) | *p=0.94 p=0.98* |
| Age   19.1-37.9  >37.9-45.9  >45.9-53.9  >53.9-84  (Missing) | 405 (24.9%) 401 (24.7%) 394 (24.2%)  393 (24.2%)  (34 (2.1%)) | 203 (24.2%) 207 (24.7%) 214 (25.5%)  215 (25.6%)  (0 (0)) | *p=0.51*  *p=0.67*  *p=0.95*  *p=0.98* |
| Highest level of education  Secondary School or less  Vocational training   Bachelor’s degree   Postgraduate degree  (Missing) | 447 (27.6%) 286 (17.7%) 554 (34.3%) 330 (20.4%) (10 (0.6%)) | 156 (18.7%) 110 (13.2%) 333 (39.9%) 236 (28.3%) (4 (0.5%)) | *p=0.50* ***p<0.001 p<0.001*** *p=0.82* |
| MS Type   Benign/RRMS  SPMS  PPMS  PRMS  Unsure/other  (Missing) | 1005 (61.8%) 197 (12.1%) 119 (7.3%) 37 (2.3%) 229 (14.1%) (40 (2.5%)) | 586 (69.9%) 78 (9.3%) 56 (6.7%) 11 (1.3%) 101 (12.0%) (7 (0.8%)) | ***p=0.007*** *p=0.208 p=0.052* ***p=0.032*** ***p=0.004*** |
| Disability (P-MSSS)  Normal/mild  Moderate  Severe  (Missing) | 792 (48.7%) 397 (24.4%) 280 (17.2%) (158 (9.7%)) | 546 (65.1%) 180 (21.5%) 90 (10.7%) 23 (2.7%) | ***p<0.001 p<0.001 p<0.001*** |
| Clinically significant fatigue   No  Yes   (Missing) | 656 (48.1%)  709 (51.9%)  (262 (16.1%)) | 475 (61.5%)  298 (38.6%)  (66 (7.9%)) | ***p<0.001 p<0.001*** |
| Taking immunomodulatory medication | |  | |
| No  Yes | 880 (54.1%) 747 (45.9%) | 441 (52.6%) 398 (47.4%) | *p=0.47* |
| Antidepressant medication prescription | |  | |
| No  Yes | 1251 (76.9%) 376 (23.1%) | 713 (85.0%) 126 (15.0%) | ***p<0.001*** |
| Anxiolytic medication prescription | |  | |
| No  Yes | 1429 (87.8%) 198 (12.2%) | 782 (93.2%) 57 (6.8%) | ***p<0.001*** |
| Taking vitamin D supplementation | |  | |
| No  Yes | 478 (29.4%) 1149 (70.6%) | 123 (14.7%) 716 (85.3%) | ***p<0.001*** |
| Number of co-morbidities  0  1  2  3+ | 863 (53.0%) 377 (23.2%) 214 (13.2%) 173 (10.6%) | 512 (61.0%) 197 (23.5%) 94 (11.2%) 36 (4.3%) | *p=0.23*  ***p=0.027 p<0.001*** |
| Depression risk (PHQ-2)  No  Yes | 1073 (75.8%)  343 (24.2%) | 726 (89.9%) 82 (10.2%) | ***p<0.001*** |
| Smoking status  Never smoked  Ex-smoker  Current smoker  (Missing) | 638 (39.2%) 615 (37.8%) 219 (13.5%) (155 (9.5%)) | 468 (55.8%) 286 (34.1%) 62 (7.4%) (23 (2.7%)) | ***p<0.001 p<0.001 p<0.001*** |
| Alcohol consumption  No intake  Limited   Heavy  (Missing) | 697 (42.8%) 582 (35.8%) 165 (10.1%) (183 (11.3%)) | 297 (35.4%) 388 (46.3%) 121 (14.4%) (33 (3.9%)) | ***p<0.001 p<0.001 p<0.001*** |
| IPAQ  Inactive  Minimally active  Active  (Missing) | 505 (31.0%) 503 (30.9%) 335 (20.6%) (284 (17.5%)) | 242 (28.4%) 347 (41.4%) 198 (23.6%)  (52 (6.2%)) | ***p=0.001***  *p=0.078*  ***p<0.001*** |
| BMI  Underweight/normal  Overweight  Obese   (Missing) | 853 (52.5%) 382 (23.5) 368 (22.6%) (24 (1.5%)) | 547 (65.2%) 175 (20.9%) 117 (13.9%) (0) | *p=0.14*  ***p=0.002***  *p=0.97* |
| Consumes meat  No  Yes | 385 (26.1%) 1090 (73.9%) | 376 (45.9%)  443 (54.1%) | ***p<0.001*** |
| Consumes dairy  No  Yes | 461 (31.5%) 1004 (68.3%) | 401 (49.4%) 411 (50.6%) | ***p<0.001*** |
|  | Mean (standard deviation) | |  |
| DHQ total score | 76.34 (12.33) | 82.85 (11.03) | ***p<0.001*** |
| HSU total score | 0.65 (0.13) | 0.71 (0.12) | ***p<0.001*** |
| Abbreviations: BMI: Body mass index; FSS: Fatigue Severity Scale; P-MSSS: Patient Determined Multiple Sclerosis Severity Score; PPMS: Primary progressive multiple sclerosis; PRMS: Progressive-relapsing multiple sclerosis; RRMS: Relapsing-remitting multiple sclerosis; SPMS: Secondary progressive multiple sclerosis; PHQ-2: Patient health questionnaire-2; IPAQ: International Physical Activity Questionnaire; HSU: Health state utility; DHQ: Diet Habits Questionnaire.  Differences between dichotomous and polychotomous variables were assessed by multinomial logistic regression. Differences between normally distributed continuous terms were assessed by two-tailed t-test.  Note: Missing proportions are additional on top of proportions with data on each parameter. Thus, total proportions add up to greater than 100%   Note: Boldface denotes significance (p<0.05).  Note: limited alcohol consumption is classified as 1 standard drink a day for females and 2 standard drink a day for males; heavy alcohol consumption is classified as >1 standard drink a day for females and >2 standard drinks a day for males. | | | |

*Supplemental Table 2. Cross-sectional characteristics of Dietary Habits Questionnaire and Health State Utilities at 2.5 and 5-year*

|  | 2.5-year | | | 5-year | | |
| --- | --- | --- | --- | --- | --- | --- |
|  | n (%) | aβ (95%CI)^a^ | aβ (95%CI)^b^ | n (%) | aβ (95%CI)^a^ | aβ (95%CI)^b^ |
| DHQ total score, 10-unit continuous |  | **0.029 (0.022, 0.036)**  ***p<0.001*** | **0.012 (0.006, 0.018)**  ***p<0.001*** |  | **0.029 (0.021, 0.037)**  ***p<0.001*** | **0.009 (0.003, 0.016)**  ***p=0.003*** |
| <75  >75-85  >85-92  >92-100 *Trend* | 236 (31.1%) 185 (24.4%) 202 (26.6%) 136 (17.9 %)   \|  \| \| --- \| | 0.00 [Ref] **0.04 (0.01, 0.05)** **0.05 (0.03, 0.08)** **0.11 (0.09, 0.14)** ***p<0.001*** | 0.00 [Ref] 0.00 (-0.02, 0.02) **0.02 (0.00, 0.04) 0.06 (0.04, 0.08) *p<0.001*** | 300 (37.4%) 244 (30.4%) 216 (26.9%) 43 (5.4%) | 0.00 [Ref] **0.04 (0.02, 0.06) 0.07 (0.05, 0.09)** **0.10 (0.06, 0.14) *p<0.001*** | 0.00 [Ref] 0.01 (-0.01, 0.03) **0.02 (0.01, 0.04) 0.04 (0.01, 0.07) *p=0.002*** |
| DHQ fat score, continuous |  | **0.050 (0.035, 0.064)**  ***p<0.001*** | **0.019 (0.008, 0.031)**  ***p=0.001*** |  | **0.045 (0.031, 0.059)**  ***p<0.001*** | **0.013 (0.003, 0.024)**  ***p=0.015*** |
| <3.7  >3.7-4.2  >4.2-4.7  >4.7-5 *Trend* | 244 (32.2%) 203 (26.8%) 159 (21.0%) 153 (20.2%) | 0.00 [Ref] **0.03 (0.01, 0.06) 0.05 (0.03, 0.08)** **0.09 (0.07, 0.12) *p<0.001*** | 0.00 [Ref] 0.01 (-0.00, 0.03) 0.02 (-0.00, 0.04) **0.05 (0.03, 0.07) *p<0.001*** | 303 (37.7%) 262 (32.6%) 148 (18.4%) 90 (11.2%) | 0.00 [Ref] **0.04 (0.03, 0.07**) **0.07 (0.05, 0.10)** **0.07 (0.04, 0.10) *p<0.001*** | 0.00 [Ref] **0.02 (0.00, 0.04)** **0.02 (0.00, 0.04) 0.03 (0.01, 0.05)** ***p=0.003*** |
| DHQ cereal score, continuous |  | **0.026 (0.017, 0.035)**  ***p<0.001*** | **0.016 (0.009, 0.023)**  ***p<0.001*** |  | **0.025 (0.016, 0.033)**  ***p<0.001*** | **0.010 (0.003, 0.017)**  ***p=0.004*** |
| <3  >3-4  >4-4.3  >4.3-5 *Trend* | 217 (28.6%) 259 (34.2%) 135 (17.8%) 147 (19.4%) | 0.00 [Ref] **0.02 (0.00, 0.05) 0.03 (0.01, 0.06)** **0.08 (0.06, 0.11) *p<0.001*** | 0.00 [Ref] 0.01 (-0.01, 0.02) 0.01 (-0.01, 0.03) **0.05 (0.03, 0.07) *p<0.001*** | 228 (28.4%) 293 (36.5%) 120 (14.9%) 162 (20.2%) | 0.00 [Ref] **0.04 (0.01, 0.06)** **0.05 (0.02, 0.08) 0.07 (0.04, 0.09)** ***p<0.001*** | 0.00 [Ref] 0.01 (-0.01, 0.03) **0.02 (0.00, 0.04) 0.02 (0.01, 0.04)** ***p=0.006*** |
| DHQ fruit and vegetable score, continuous |  | **0.032 (0.021, 0.043)**  ***p<0.001*** | **0.009 (0.000, 0.018)**  ***p=0.041*** |  | **0.030 (0.020, 0.041)**  ***p<0.001*** | 0.006 (-0.003, 0.014)  *p=0.17* |
| <3.2  >3.2-4  >4-4.4  >4.4-5 *Trend* | 202 (26.6%) 232 (30.6%) 163 (21.5%) 162 (21.3%) | 0.00 [Ref] 0.02 (-0.01, 0.04) **0.04 (0.01, 0.06)** **0.07 (0.04, 0.09) *p<0.001*** | 0.00 [Ref] -0.01 (-0.02, 0.01) 0.01 (-0.01, 0.03) **0.02 (0.00, 0.04)** ***p=0.021*** | 209 (26.0%) 252 (31.3%) 153 (19.0%) 190 (23.6%) | 0.00 [Ref] **0.04 (0.02, 0.06) 0.05 (0.03, 0.08) 0.06 (0.04, 0.09)** ***p<0.001*** | 0.00 [Ref] 0.00 (-0.01, 0.02) 0.01 (-0.00, 0.03) 0.01 (-0.00, 0.03) *p=0.10* |
| DHQ takeaway score, continuous |  | **0.024 (0.013, 0.035)**  ***p<0.001*** | 0.007 (-0.002, 0.016)  *p=0.12* |  | 0.002 (-0.011, 0.015)  *p=0.80* | -0.001 (-0.011, 0.009)  *p=0.84* |
| <3.7  >3.7-4.3  >4.3-5 *Trend* | 211 (28.9%) 196 (26.8%) 324 (44.3%) | 0.00 [Ref] 0.01 (-0.01, 0.03) **0.05 (0.03, 0.07) *p<0.001*** | 0.00 [Ref] 0.00 (-0.02, 0.02) **0.02 (0.00, 0.04)** ***p=0.021*** | 366 (73.2%) 66 (13.2%) 68 (13.6%) | 0.00 [Ref] 0.01 (-0.02, 0.04) -0.01 (-0.04, 0.03) *p=0.83* | 0.00 [Ref] -0.00 (-0.03, 0.02) -0.00 (-0.02, 0.02) *p=0.94* |
| DHQ food choices score, continuous |  | **0.030 (0.020, 0.040)**  ***p<0.001*** | **0.010 (0.002, 0.018)**  ***p=0.010*** |  | **0.028 (0.019, 0.037)**  ***p<0.001*** | **0.008 (0.000, 0.015)**  ***p=0.037*** |
| <3.8  >3.8-4.5  >4.5-5 *Trend* | 231 (30.9%) 201 (26.9%) 315 (42.2%) | 0.00 [Ref] **0.05 (0.03, 0.07)** **0.08 (0.05, 0.10) *p<0.001*** | 0.00 [Ref] 0.01 (-0.00, 0.03) **0.03 (0.02, 0.05)** ***p<0.001*** | 272 (34.2%) 236 (29.7%) 287 (36.1%) | 0.00 [Ref] **0.04 (0.01, 0.06) 0.06 (0.04, 0.08)** ***p<0.001*** | 0.00 [Ref] 0.01 (-0.01, 0.02) **0.02 (0.00, 0.03) *p=0.021*** |
| DHQ omega-3 score, continuous |  | **0.012 (0.006, 0.019)**  ***p<0.001*** | 0.001 (-0.004, 0.007)  *p=0.57* |  | **0.010 (0.004, 0.017)**  ***p=0.001*** | 0.003 (-0.002, 0.008)  *p=0.18* |
| <3  >3-4 >4-5  *Trend* | 316 (41.7%) 187 (24.7%) 254 (33.6%) | 0.00 [Ref] **0.03 (0.00, 0.05) 0.04 (0.02, 0.06) *p<0.001*** | 0.00 [Ref] 0.01 (-0.02, 0.02) 0.01 (-0.01, 0.02) *p=0.33* | 360 (44.3%) 189 (23.5%) 254 (31.6%) | 0.00 [Ref] **0.02 (0.00, 0.04) 0.04 (0.02, 0.06)**  ***p<0.001*** | 0.00 [Ref] 0.01 (-0.01, 0.02) 0.01 (-0.00, 0.03) *p=0.12* |
| DHQ food preparation score, continuous |  | **0.038 (0.024, 0.053)**  ***p<0.001*** | **0.017 (0.006, 0.029)**  ***p=0.003*** |  | **0.035 (0.021, 0.049)**  ***p<0.001*** | **0.012 (0.001, 0.023)**  ***p=0.030*** |
| <4.4  >4.4-5 | 261 (35.1%) 482 (64.9%) | 0.00 [Ref] **0.05 (0.03, 0.07) *p<0.001*** | 0.00 [Ref] **0.02 (0.01, 0.04)** ***p=0.001*** | 300 (38.0%) 489 (62.0%) | 0.00 [Ref] **0.04 (0.02, 0.06)** ***p<0.001*** | 0.00 [Ref] **0.01 (0.00, 0.03) *p=0.049*** |
| DHQ fibre score, continuous |  | **0.042 (0.030, 0.054)**  ***p<0.001*** | **0.018 (0.008, 0.028)**  ***p<0.001*** |  | **0.038 (0.026, 0.049)**  ***p<0.001*** | **0.011 (0.002, 0.021)**  ***p=0.016*** |
| <3.3  >3.3-3.9  >3.9-4.4  >4.4-5 *Trend* | 196 (26.6%) 194 (26.3%) 207 (28.1%) 140 (19.0%) | 0.00 [Ref] 0.01 (-0.01, 0.04) **0.05 (0.03, 0.07)** **0.09 (0.07, 0.12) *p<0.001*** | 0.00 [Ref] -0.01 (-0.03, 0.01) 0.01 (-0.00, 0.03) **0.05 (0.02, 0.07)** ***p<0.001*** | 216 (27.7%) 205 (26.3%) 200 (25.6%) 160 (20.5%) | 0.00 [Ref] **0.04 (0.02, 0.06) 0.06 (0.03, 0.08)** **0.07 (0.05, 0.10) *p<0.001*** | 0.00 [Ref] 0.02 (-0.00, 0.04) 0.02 (-0.00, 0.03) **0.03 (0.01, 0.05)** ***p=0.007*** |
| Consumes meat   No  Yes | 350 (46.1%) 409 (53.9%) | 0.00 [Ref] **-0.05 (-0.07, -0.04) *p<0.001*** | 0.00 [Ref] **-0.02 (-0.04, -0.01)** ***p=0.003*** | 363 (45.2%) 440 (54.8%) | 0.00 [Ref] **-0.06 (-0.07, -0.04) *p<0.001*** | 0.00 [Ref] **-0.02 (-0.03, -0.01)** ***p=0.004*** |
| Consumes dairy  No  Yes | 373 (49.2%) 385 (50.8%) | 0.00 [Ref] **-0.05 (-0.07, -0.03) *p<0.001*** | 0.00 [Ref] **-0.02 (-0.03, -0.01)** ***p=0.008*** | 388 (48.4%) 414 (51.6%) | 0.00 [Ref] **-0.06 (-0.07, -0.04) *p<0.001*** | 0.00 [Ref] **-0.02 (-0.03, -0.01)** ***p=0.007*** |
| Abbreviations: aβ= adjusted Beta coefficient; n: Number of participants; DHQ: Diet Habits Questionnaire  Analysis by lagged panel-data linear regression, estimating β (95% confidence intervals (CI))  Note: Boldface denotes significance (p<0.05).  ^a^ Model 1 adjusted for experiencing ongoing symptoms due to relapse  ^b^ Model 2 further adjusted for age, sex, level of highest education, disability (P-MSSS), clinically significant fatigue, prescription antidepressant medication use, treated comorbidity number and depression-risk (PHQ-2). | | | | | | |

*Supplemental Table 3. Separate 2.5-year prospective analyses of DHQ-HSU relationships baseline-2.5 and 2.5-5-year*

| Category | aβ (95% CI)^a^ (Panel regression) | aβ (95% CI)^b^  (Baseline to 2.5 years) | aβ (95% CI)^b^ (2.5 to 5-years) |
| --- | --- | --- | --- |
| DHQ total score, 10-unit continuous | **0.010 (0.005, 0.014)**  ***p<0.001*** | **0.010 (0.003, 0.016)**  ***p=0.003*** | **0.009 (0.003, 0.015)**  ***p=0.004*** |
| <75  >75-85  >85-92  >92-100 *Trend* | 0.00 [Ref] 0.01 (-0.00, 0.02) **0.02 (0.01, 0.04)** **0.03 (0.01, 0.04)** ***p<0.001*** | 0.00 [Ref] 0.01 (-0.01, 0.03) 0.02 (-0.00, 0.04) **0.03 (0.01, 0.05)** ***p=0.007*** | 0.00 [Ref] -0.00 (-0.02, 0.02)  **0.02 (0.00, 0.04) 0.04 (0.02, 0.06)** ***p<0.001*** |
| DHQ fat score, continuous | **0.016 (0.007, 0.025)**  ***p<0.001*** | **0.017 (0.004, 0.030)**  ***p=0.009*** | **0.013 (0.002, 0.025)**  ***p=0.022*** |
| <3.7  >3.7-4.2  >4.2-4.7  >4.7-5 *Trend* | 0.00 [Ref] 0.01 (-0.00, 0.02) **0.01 (0.00, 0.03) 0.02 (0.01, 0.04) *p=0.001*** | 0.00 [Ref] **0.03 (0.01, 0.04)** 0.01 (-0.01, 0.03) **0.03 (0.01, 0.05) *p=0.022*** | 0.00 [Ref] -0.01 (-0.02, 0.01)  0.01 (-0.01, 0.03) **0.03 (0.01, 0.05) *p=0.001*** |
| DHQ cereal score, continuous | **0.007 (0.002, 0.013)**  ***p=0.009*** | 0.006 (-0.002, 0.014)  *p=0.12* | **0.012 (0.005, 0.019)**  ***p=0.001*** |
| <3  >3-4  >4-4.3  >4.3-5 Trend | 0.00 [Ref] **0.01 (0.00, 0.03) 0.02 (0.00, 0.03)** **0.02 (0.01, 0.04) *p=0.004*** | 0.00 [Ref] **0.02 (0.00, 0.04)** 0.01 (-0.01, 0.04) 0.02 (0.00, 0.04) *p=0.057* | 0.00 [Ref] 0.01 (-0.01, 0.03) 0.02 (-0.00, 0.04) **0.03 (0.01, 0.05)** ***p=0.001*** |
| DHQ fruit and vegetable score, continuous | **0.010 (0.003, 0.016)**  ***p=0.004*** | **0.011 (0.002, 0.020)**  ***p=0.012*** | 0.006 (-0.003, 0.015)  *p=0.18* |
| <3.2  >3.2-4  >4-4.4  >4.4-5  Trend | 0.00 [Ref] 0.00 (-0.01, 0.01) **0.02 (0.01, 0.04)** **0.02 (0.00, 0.03) *p=0.001*** | 0.00 [Ref] 0.01 (-0.01, 0.03) 0.01 (-0.01, 0.03) **0.03 (0.01, 0.05)** ***p=0.016*** | 0.00 [Ref] -0.01 (-0.03, 0.01)  **0.02 (0.00, 0.04)** 0.01 (-0.01, 0.03) ***p=0.035*** |
| DHQ takeaway score, continuous | **0.010 (0.003, 0.016)**  ***p=0.004*** | 0.008 (-0.001, 0.017)  *p=0.079* | **0.011 (0.002, 0.019)**  ***p=0.012*** |
| <3.7  >3.7-4.3  >4.3-5 *Trend* | 0.00 [Ref] 0.01 (-0.01, 0.02) **0.02 (0.00, 0.03) *p=0.014*** | 0.00 [Ref] -0.00 (-0.02, 0.02) 0.01 (-0.01, 0.03) *p=0.30* | 0.00 [Ref] 0.01 (-0.00, 0.03) **0.03 (0.01, 0.04)**  ***p=0.003*** |
| DHQ food choices score, continuous | **0.009 (0.002, 0.015)**  ***p=0.006*** | **0.012 (0.003, 0.021)**  ***p=0.008*** | 0.006 (-0.002, 0.014)  *p=0.12* |
| <3.8  >3.8-4.5  >4.5-5  Trend | 0.00 [Ref] 0.00 (-0.01, 0.02) **0.02 (0.01, 0.03) *p=0.001*** | 0.00 [Ref] 0.01 (-0.01, 0.03) **0.02 (0.01, 0.04)** ***p=0.009*** | 0.00 [Ref] -0.01 (-0.02, 0.01) **0.02 (0.00, 0.04) *p=0.007*** |
| DHQ omega-3 score, continuous | 0.004 (-0.000, 0.007)  *p=0.070* | 0.003 (-0.002, 0.009)  *p=0.25* | 0.003 (-0.002, 0.009)  *p=0.18* |
| <3  >3-4  >4-5  Trend | 0.00 [Ref] 0.01 (-0.00, 0.02) **0.02 (0.01, 0.03) *p=0.005*** | 0.00 [Ref] -0.01 (-0.02, 0.01)  **0.02 (0.00, 0.03)** ***p=0.037*** | 0.00 [Ref] 0.01 (-0.01, 0.03) 0.01 (-0.00, 0.03) *p=0.67* |
| DHQ food preparation score, continuous | **0.011 (0.002, 0.019)**  ***p=0.015*** | 0.010 (-0.003, 0.023)  *p=0.13* | 0.011 (-0.001, 0.022)  *p=0.065* |
| <4.4  >4.4-5 | 0.00 [Ref] 0.01 (-0.00, 0.02) *p=0.15* | 0.00 [Ref] 0.01 (-0.00, 0.03) *p=0.13* | 0.00 [Ref] 0.01 (-0.00, 0.02) *p=0.21* |
| DHQ fibre score, continuous | **0.012 (0.005, 0.020)**  ***p=0.001*** | **0.013 (0.003, 0.023)**  ***p=0.009*** | **0.013 (0.003, 0.023)**  ***p=0.013*** |
| <3.3  >3.3-3.9  >3.9-4.4  >4.4-5 Trend | 0.00 [Ref] **0.02 (0.01, 0.03)** **0.02 (0.01, 0.03) 0.03 (0.01, 0.04) *p=0.002*** | 0.00 [Ref] **0.02 (0.00, 0.04) 0.02 (0.00, 0.04) 0.03 (0.01, 0.05)** ***p=0.010*** | 0.00 [Ref] 0.01 (-0.01, 0.03) 0.01 (-0.00, 0.03) **0.03 (0.01, 0.05)** ***p=0.006*** |
| Consumes meat   No  Yes | 0.00 [Ref] **-0.02 (-0.03, -0.01) *p=0.001*** | 0.00 [Ref] -0.01 (-0.03, 0.00) *p=0.070* | 0.00 [Ref] -**0.02 (-0.04, -0.01)** ***p=0.001*** |
| Consumes dairy  No  Yes | 0.00 [Ref] **-0.01 (-0.02, -0.00) *p=0.008*** | 0.00 [Ref] **-0.02 (-0.03, -0.01)** ***p=0.007*** | 0.00 [Ref] -0.01 (-0.03, 0.00) *p=0.051* |
| Abbreviations: aβ= Adjusted Beta coefficient; HSU: Health state utility; DHQ: Diet Habits Questionnaire.  Note: Boldface denotes significance (p<0.05).  Model ^a^ longitudinal panel linear regression analysis (baseline vs 2.5 and 2.5 vs 5-years)  Model ^b^ separate baseline vs 2.5-year and 2.5-year vs 5-year analyses (baseline DHQ *predicted* 2.5 HSU and 2.5 DHQ *predicted* 5-years HSU)  Model adjusted for experiencing ongoing symptoms due to relapse, age, sex, level of highest education, disability (P-MSSS), clinically significant fatigued, prescription antidepressant medication use, treated comorbidity number, depression-risk (PHQ-2) and baseline HSU. | | | |

*Supplemental Table 4. Prospective associaions between lagged diet parameters and total SF-6D HSU and subdomains (adjusted difference in medians (95% CI)*

| **SF-6D score** | **Total HSU** | **Physical health** | **Role limitation** | **Social functioning** |  |
| --- | --- | --- | --- | --- | --- |
| DHQ total score, 10-unit continuous | **0.010 (0.005, 0.014)**  ***p<0.001*** | **0.015 (0.008, 0.023)**  ***p<0.001*** | 0.010 (-0.001, 0.022)  *p=0.085* | 0.004 (-0.004, 0.012)  *p=0.36* |  |
| <75   >75-85   >85-92   >92-100 *Trend* | 0.00 [Reference]  0.01 (-0.01, 0.02)  **0.02 (0.01, 0.03)**  **0.03 (0.02, 0.05)**  ***p<0.001*** | 0.00 [Reference]  **0.03 (0.01, 0.05)**  **0.04 (0.02, 0.07)**  **0.04 (0.02, 0.07)**  ***p<0.001*** | 0.00 [Reference]  0.03 (-0.01, 0.07)  **0.04 (0.00, 0.07)**  0.03 (-0.00, 0.07)  ***p=0.049*** | 0.00 [Reference]  -0.00 (-0.03, 0.03)  0.01 (-0.01, 0.04)  0.01 (-0.02, 0.04)  *p=0.30* |  |
| DHQ fat score, continuous | **0.016 (0.008, 0.024)**  ***p<0.001*** | **0.029 (0.014, 0.044)**  ***p<0.001*** | 0.019 (-0.003, 0.042)  *p=0.093* | 0.005 (-0.010, 0.021)  *p=0.49* |  |
| <3.7   >3.7-4.2   >4.2-4.7   >4.7-5 *Trend* | 0.00 [Reference]  0.01 (-0.00, 0.02)  0.01 (-0.00, 0.03)  **0.03 (0.02, 0.04)**  ***p<0.001*** | 0.00 [Reference]  **0.03 (0.01, 0.05)**  0.02 (-0.00, 0.05)  **0.05 (0.02, 0.07)**  ***p<0.001*** | 0.00 [Reference]  -0.01 (-0.04, 0.03)  0.01 (-0.02, 0.05)  0.03 (-0.00, 0.06)  *p=0.063* | 0.00 [Reference]  0.01 (-0.02, 0.03)  0.01 (-0.01, 0.04)  0.01 (-0.01, 0.04)  *p=0.28* |  |
| DHQ cereal score, continuous | **0.009 (0.004, 0.014)**  ***p=0.001*** | 0.009 (-0.001, 0.018)  *p=0.072* | 0.004 (-0.009, 0.017)  *p=0.56* | 0.006 (-0.004, 0.016)  *p=0.24* |  |
| <3   >3-4   >4-4.3   >4.3-5 *Trend* | 0.00 [Reference]  **0.02 (0.00, 0.03)**  **0.02 (0.00, 0.03)**  **0.03 (0.01, 0.04)**  ***p<0.001*** | 0.00 [Reference]  0.01 (-0.01, 0.04)  **0.02 (-0.01, 0.05)**  0.01 (-0.02, 0.03)  *p=0.47* | 0.00 [Reference]  0.01 (-0.02, 0.05)  0.01 (-0.03, 0.05)  0.02 (-0.02, 0.05)  *p=0.38* | 0.00 [Reference]  0.02 (-0.01, 0.04)  -0.00 (-0.03, 0.03)  0.02 (-0.01, 0.04)  *p=0.39* |  |
| DHQ fruit and vegetable score, continuous | **0.009 (0.003, 0.015)**  ***p=0.004*** | **0.014 (0.003, 0.025)**  ***p=0.013*** | 0.010 (-0.006, 0.026)  *p=0.23* | 0.003 (-0.008, 0.015)  *p=0.58* |  |
| <3.2   >3.2-4   >4-4.4   >4.4-5 *Trend* | 0.00 [Reference]  -0.00 (-0.01, 0.01)  **0.02 (0.00, 0.03)**  **0.02 (0.00, 0.03)**  ***p=0.001*** | 0.00 [Reference]  0.00 (-0.02, 0.03)  **0.04 (0.01, 0.06)**  0.02 (-0.00, 0.05)  ***p=0.012*** | 0.00 [Reference]  0.01 (-0.02, 0.04)  0.02 (-0.02, 0.05)  0.02 (-0.02, 0.06)  *p=0.22* | 0.00 [Reference]  -0.01 (-0.04, 0.01)  0.00 (-0.02, 0.03)  0.01 (-0.02, 0.03)  *p=0.42* |  |
| DHQ takeaway score, continuous | **0.010 (0.004, 0.016)**  ***p=0.001*** | 0.011 (-0.000, 0.021)  *p=0.052* | 0.009 (-0.007, 0.025)  *p=0.26* | 0.001 (-0.010, 0.012)  *p=0.89* |  |
| <3.7   >3.7-4.3   >4.3-5 *Trend* | 0.00 [Reference]  0.01 (-0.00, 0.02)  **0.02 (0.01, 0.03)**  ***p=0.002*** | 0.00 [Reference]  0.01 (-0.01, 0.04)  0.02 (-0.00, 0.04)  *p=0.067* | 0.00 [Reference]  0.01 (-0.02, 0.04)  0.02 (-0.01, 0.05)  *p=0.21* | 0.00 [Reference]  0.01 (-0.02 ,0.03)  -0.00 (-0.02, 0.02)  *p=0.90* |  |
| DHQ food choices score, continuous | **0.009 (0.003, 0.015)**  ***p=0.002*** | **0.019 (0.009, 0.029)**  ***p<0.001*** | 0.006 (-0.009, 0.021)  *p=0.45* | 0.005 (-0.006, 0.016)  *p=0.38* |  |
| <3.8   >3.8-4.5   >4.5-5 *Trend* | 0.00 [Reference]  0.00 (-0.01, 0.02)  **0.02 (0.01, 0.03)**  ***p<0.001*** | 0.00 [Reference]  **0.02 (0.00, 0.05)**  **0.04 (0.02, 0.06)**  ***p=0.001*** | 0.00 [Reference]  -0.01 (-0.04, 0.03)  0.01 (-0.02, 0.04)  *p=0.36* | 0.00 [Reference]  0.01 (-0.02, 0.03)  0.01 (-0.01, 0.03)  *p=0.49* |  |
| DHQ omega-3 score, continuous | 0.004 (-0.000, 0.007)  *p=0.059* | 0.005 (-0.002, 0.011)  *p=0.15* | 0.006 (-0.003, 0.016)  *p=0.17* | -0.001 (-0.007, 0.006)  *p=0.87* |  |
| <3   >3-4   >4-5  *Trend* | 0.00 [Reference]  0.00 (-0.01, 0.02)  **0.02 (0.00, 0.03)**  ***p=0.005*** | 0.00 [Reference]  0.00 (-0.02, 0.03)  0.02 (-0.00, 0.04)  *p=0.12* | 0.00 [Reference]  0.00 (-0.03, 0.03)  0.02 (-0.00, 0.05)  *p=0.086* | 0.00 [Reference]  0.01 (-0.01, 0.04)  -0.00 (-0.02, 0.02)  *p=0.80* |  |
| DHQ food preparation score, continuous | **0.011 (0.003, 0.019)**  ***p=0.009*** | **0.025 (0.009, 0.041)**  ***p=0.002*** | **0.025 (0.001, 0.049)**  ***p=0.037*** | 0.006 (-0.010, 0.022)  *p=0.44* |  |
| <4.4   >4.4-5 | 0.00 [Reference]  **0.01 (0.00, 0.02)**  ***p=0.040*** | 0.00 [Reference]  **0.02 (0.01, 0.04)**  ***p=0.011*** | 0.00 [Reference]  **0.03 (0.00, 0.06)**  ***p=0.040*** | 0.00 [Reference]  0.01 (-0.01, 0.03)  *p=0.51* |  |
| DHQ fibre score, continuous | **0.013 (0.006, 0.020)**  ***p<0.001*** | **0.017 (0.004, 0.030)**  ***p=0.008*** | 0.007 (-0.011, 0.025)  *p=0.47* | 0.007 (-0.006, 0.020)  *p=0.31* |  |
| <3.3   >3.3-3.9   >3.9-4.4   >4.4-5 *Trend* | 0.00 [Reference]  **0.02 (0.00, 0.03)**  **0.02 (0.00, 0.03)**  **0.03 (0.02, 0.04)**  ***p<0.001*** | 0.00 [Reference]  0.02 (-0.00, 0.05)  0.02 (-0.00, 0.05)  **0.03 (0.01, 0.06)**  ***p=0.026*** | 0.00 [Reference]  0.02 (-0.01, 0.06)  0.01 (-0.02, 0.05)  0.02 (-0.02, 0.05)  *p=0.50* | 0.00 [Reference]  0.01 (-0.01, 0.04)  0.01 (-0.02, 0.03)  0.02 (-0.01, 0.05)  *p=0.29* |  |
| Consumes meat   No   Yes | 0.00 [Reference]  **-0.02 (-0.03, -0.01)**  ***p<0.001*** | 0.00 [Reference]  **-0.03 (-0.05, -0.02)**  ***p<0.001*** | 0.00 [Reference]  -0.02 (-0.04, 0.01)  *p=0.14* | 0.00 [Reference]  -0.01 (-0.03, 0.01)  *p=0.19* |  |
| Consumes dairy   No    Yes | 0.00 [Reference]  **-0.02 (-0.03, -0.01)**  ***p=0.001*** | 0.00 [Reference]  -0.02 (-0.03, 0.00)  *p=0.089* | 0.00 [Reference]  -0.02 (-0.04, 0.01)  *p=0.13* | 0.00 [Reference]  -0.01 (-0.03, 0.00)  *p=0.15* |  |
| **SF-6D score** | **Total HSU** | **Pain** | **Mental health** | **Vitality** |  |
| DHQ total score, 10-unit continuous | **0.010 (0.005, 0.014)**  ***p<0.001*** | **0.022 (0.014, 0.031)**  ***p<0.001*** | 0.005 (-0.003, 0.014)  *p=0.23* | **0.014 (0.006, 0.023)**  ***p=0.001*** |  |
| <75   >75-85   >85-92   >92-100 *Trend* | 0.00 [Reference]  0.01 (-0.01, 0.02)  **0.02 (0.01, 0.03)**  **0.03 (0.02, 0.05)**  ***p<0.001*** | 0.00 [Reference]  **0.03 (0.00, 0.06)**  **0.04 (0.02, 0.07)**  **0.08 (0.06, 0.11)**  ***p<0.001*** | 0.00 [Reference]  -0.01 (-0.04, 0.02)  0.00 (-0.02, 0.03)  0.03 (-0.00, 0.05)  *p=0.055* | 0.00 [Reference]  0.01 (-0.01, 0.04)  **0.03 (0.00, 0.05)**  **0.05 (0.02, 0.07)**  ***p=0.001*** |  |
| DHQ fat score, continuous | **0.016 (0.008, 0.024)**  ***p<0.001*** | **0.044 (0.028, 0.061)**  ***p<0.001*** | 0.006 (-0.011, 0.022)  *p=0.49* | **0.018 (0.002, 0.034)**  ***p=0.030*** |  |
| <3.7   >3.7-4.2   >4.2-4.7   >4.7-5 *Trend* | 0.00 [Reference]  0.01 (-0.00, 0.02)  0.01 (-0.00, 0.03)  **0.03 (0.02, 0.04)**  ***p<0.001*** | 0.00 [Reference]  **0.03 (0.00, 0.06)**  **0.06 (0.03, 0.08)**  **0.07 (0.05, 0.10)**  ***p<0.001*** | 0.00 [Reference]  -0.01 (-0.03, 0.02)  -0.02 (-0.04, 0.01)  0.02 (-0.00, 0.05)  *p=0.19* | 0.00 [Reference]  0.01 (-0.01, 0.04)  0.00 (-0.02, 0.03) **0.04 (0.02, 0.07)**  ***p=0.003*** |  |
| DHQ cereal score, continuous | **0.009 (0.004, 0.014)**  ***p=0.001*** | **0.018 (0.007, 0.029)**  ***p=0.001*** | 0.003 (-0.007, 0.013)  *p=0.61* | **0.012 (0.002, 0.022)**  ***p=0.022*** |  |
| <3   >3-4   >4-4.3   >4.3-5 *Trend* | 0.00 [Reference]  **0.02 (0.00, 0.03)**  **0.02 (0.00, 0.03)**  **0.03 (0.01, 0.04)**  ***p<0.001*** | 0.00 [Reference]  **0.04 (0.01, 0.06)**  **0.04 (0.01, 0.07)**  **0.05 (0.02, 0.08)**  ***p<0.001*** | 0.00 [Reference]  -0.01 (-0.04, 0.01)  -0.01 (-0.04, 0.02)  0.02 (-0.00, 0.05)  *p=0.066* | 0.00 [Reference]  0.02 (-0.00, 0.05)  0.03 (-0.00, 0.06)  **0.04 (0.01, 0.07)**  ***p=0.005*** |  |
| DHQ fruit and vegetable score, continuous | **0.009 (0.003, 0.015)**  ***p=0.004*** | **0.014 (0.001, 0.026)**  ***p=0.029*** | **0.012 (0.000, 0.024)**  ***p=0.044*** | **0.026 (0.014, 0.037)**  ***p<0.001*** |  |
| <3.2   >3.2-4   >4-4.4   >4.4-5 *Trend* | 0.00 [Reference]  -0.00 (-0.01, 0.01)  **0.02 (0.00, 0.03)**  **0.02 (0.00, 0.03)**  ***p=0.001*** | 0.00 [Reference]  -0.01 (-0.04, 0.02)  **0.03 (0.00, 0.06)**  0.02 (-0.01, 0.05)  ***p=0.018*** | 0.00 [Reference]  0.00 (-0.02, 0.03)  0.02 (-0.01, 0.05)  **0.03 (0.01, 0.06)**  ***p=0.006*** | 0.00 [Reference]  **0.03 (0.01, 0.06)**  **0.04 (0.01, 0.06)**  **0.06 (0.03, 0.09)**  ***p<0.001*** |  |
| DHQ takeaway score, continuous | **0.010 (0.004, 0.016)**  ***p=0.001*** | **0.018 (0.006, 0.029)**  ***p=0.003*** | **0.015 (0.003, 0.027)**  ***p=0.012*** | **0.014 (0.002, 0.025)**  ***p=0.020*** |  |
| <3.7   >3.7-4.3   >4.3-5 *Trend* | 0.00 [Reference]  0.01 (-0.00, 0.02)  **0.02 (0.01, 0.03)**  ***p=0.002*** | 0.00 [Reference]  0.01 (-0.01, 0.04)  **0.04 (0.01, 0.06)**  ***p=0.002*** | 0.00 [Reference]  0.02 (0.00, 0.05)  0.02 (-0.00, 0.04)  *p=0.12* | 0.00 [Reference]  0.01 (-0.02, 0.03)  **0.03 (0.01, 0.05)**  ***p=0.013*** |  |
| DHQ food choices score, continuous | **0.009 (0.003, 0.015)**  ***p=0.002*** | **0.030 (0.019, 0.041)**  ***p<0.001*** | -0.003 (-0.014, 0.008)  *p=0.61* | 0.009 (-0.002, 0.020)  *p=0.10* |  |
| <3.8   >3.8-4.5   >4.5-5 *Trend* | 0.00 [Reference]  0.00 (-0.01, 0.02)  **0.02 (0.01, 0.03)**  ***p<0.001*** | 0.00 [Reference]  0.02 (-0.00, 0.05)  **0.07 (0.05, 0.09)**  ***p<0.001*** | 0.00 [Reference]  -0.02 (-0.05, 0.00)  -0.00 (-0.02, 0.02)  *p=0.87* | 0.00 [Reference]  -0.01 (-0.03, 0.02)  **0.03 (0.00, 0.05)**  ***p=0.016*** |  |
| DHQ omega-3 score, continuous | 0.004 (-0.000, 0.007)  *p=0.059* | **0.012 (0.005, 0.019)**  ***p=0.001*** | -0.000 (-0.007, 0.007)  *p=0.97* | 0.003 (-0.004, 0.010)  *p=0.46* |  |
| <3   >3-4   >4-5  *Trend* | 0.00 [Reference]  0.00 (-0.01, 0.02)  **0.02 (0.00, 0.03)**  ***p=0.005*** | 0.00 [Reference]  0.02 (-0.01, 0.04)  **0.05 (0.03, 0.07)**  ***p<0.001*** | 0.00 [Reference]  -0.01 (-0.04, 0.01)  0.01 (-0.01, 0.03)  *p=0.42* | 0.00 [Reference]  0.01 (-0.00, 0.04)  0.01 (-0.01, 0.04)  *p=0.20* |  |
| DHQ food preparation score, continuous | **0.011 (0.003, 0.019)**  ***p=0.009*** | **0.035 (0.017, 0.052)**  ***p<0.001*** | -0.004 (-0.020, 0.012)  *p=0.64* | 0.007 (-0.010, 0.023)  *p=0.41* |  |
| <4.4   >4.4-5 | 0.00 [Reference]  **0.01 (0.00, 0.02)**  ***p=0.040*** | 0.00 [Reference]  **0.04 (0.02, 0.06)**  ***p<0.001*** | 0.00 [Reference]  -0.01 (-0.03, 0.01)  *p=0.18* | 0.00 [Reference]  0.01 (-0.01, 0.03)  *p=0.32* |  |
| DHQ fibre score, continuous | **0.013 (0.006, 0.020)**  ***p<0.001*** | **0.023 (0.009, 0.038)**  ***p=0.001*** | 0.013 (-0.000, 0.027)  *p=0.056* | **0.028 (0.014, 0.041)**  ***p<0.001*** |  |
| <3.3   >3.3-3.9   >3.9-4.4   >4.4-5 *Trend* | 0.00 [Reference]  **0.02 (0.00, 0.03)**  **0.02 (0.00, 0.03)**  **0.03 (0.02, 0.04)**  ***p<0.001*** | 0.00 [Reference]  0.00 (-0.02 ,0.03)  0.02 (-0.00, 0.05)  **0.05 (0.02, 0.08)**  ***p=0.001*** | 0.00 [Reference]  0.01 (-0.02, 0.04)  0.02 (-0.01, 0.05)  **0.03 (0.00, 0.06)**  ***p=0.023*** | 0.00 [Reference]  **0.04 (0.01, 0.06)**  **0.03 (0.01, 0.06)**  **0.05 (0.03, 0.08)**  ***p=0.001*** |  |
| Consumes meat   No   Yes | 0.00 [Reference]  **-0.02 (-0.03, -0.01)**  ***p<0.001*** | 0.00 [Reference]  **-0.05 (-0.07, -0.03)**  ***p<0.001*** | 0.00 [Reference]  -0.01 (-0.02, 0.01)  *p=0.56* | 0.00 [Reference]  **-0.02 (-0.04, -0.00)**  ***p=0.019*** |  |
| Consumes dairy   No    Yes | 0.00 [Reference]  **-0.02 (-0.03, -0.01)**  ***p=0.001*** | 0.00 [Reference]  **-0.05 (-0.07, -0.03)**  ***p<0.001*** | 0.00 [Reference]  -0.00 (-0.02, 0.01)  *p=0.63* | 0.00 [Reference]  **-0.02 (-0.04, -0.00)**  ***p=0.045*** |  |
| Abbreviations: DHQ: Dietary Habits Questionnaire; HSU: Health State Utility  Changes in dichotomised SF-6D HSU and subdomains assessed by panel-data quantile regression, estimating adjusted difference in median (β) (95%CI).  Note: Boldface denotes significance (p<0.05).  Model adjusted for experiencing ongoing symptoms due to relapse, age, sex, level of highest education, disability (P-MSSS), clinically significant fatigued, prescription antidepressant medication use, treated comorbidity number, depression-risk (PHQ-2) and baseline HSU. | | | | | |

*Supplemental Table 5. Prospective diet characteristics of dichotomised total HSU and subscores at 2.5 and 5-year reviews.*

| SF-6D score | **Total HSU** | | **Physical health** | | **Role limitation** | |
| --- | --- | --- | --- | --- | --- | --- |
|  | aβ (95% CI) (2.5 years) | aβ (95% CI) (5 years) | aβ (95% CI) (2.5 years) | aβ (95% CI) (5 years) | aβ (95% CI) (2.5 years) | aβ (95% CI) (5 years) |
| DHQ total score, 10-unit continuous | **0.008 (0.001, 0.016)**  ***p=0.025*** | 0.005 (-0.005, 0.015)  *p=0.31* | 0.011 (-0.003, 0.026)  *p=0.14* | **0.019 (0.005, 0.034)**  ***p=0.008*** | 0.002 (-0.028, 0.031)  *p=0.91* |  |
| <75   >75-85   >85-92   >92-100 *Trend* | 0.00 [Reference]  0.01 (-0.01, 0.04)  0.03 (-0.00, 0.05)  0.02 (-0.00, 0.05)  ***p=0.047*** | 0.00 [Reference]  0.01 (-0.02, 0.04)  0.01 (-0.02, 0.04)  0.03 (-0.00, 0.05)  *p=0.10* | 0.00 [Reference]  0.03 (-0.01, 0.07)  0.04 (-0.00, 0.08)  0.04 (-0.01, 0.08)  ***p=0.047*** | 0.00 [Reference]  0.03 (-0.01, 0.07)  **0.04 (0.00, 0.09)**  **0.06 (0.02, 0.10)**  ***p=0.007*** | 0.00 [Reference]  0.02 (-0.07, 0.11)  0.01 (-0.08, 0.11)  0.01 (-0.08, 0.11)  *p=0.90* |  |
| DHQ fat score, continuous | **0.015 (0.001, 0.028)**  ***p=0.033*** | 0.011 (-0.008, 0.029)  *p=0.26* | 0.023 (-0.006, 0.052)  *p=0.12* | **0.041 (0.012, 0.070)**  ***p=0.005*** | 0.0098 (-0.048, 0.067)  *p=0.75* |  |
| <3.7   >3.7-4.2   >4.2-4.7   >4.7-5 *Trend* | 0.00 [Reference]  **0.01 (-0.01, 0.03)**  0.01 (-0.01, 0.04)  **0.03 (0.01, 0.05)**  ***p=0.015*** | 0.00 [Reference]  0.01 (-0.02, 0.04)  -0.01 (-0.04, 0.02)  0.03 (-0.00, 0.05)  *p=0.16* | 0.00 [Reference]  0.04 (-0.00, 0.09)  0.01 (-0.04, 0.05)  **0.06 (0.01, 0.10)**  *p=0.053* | 0.00 [Reference]  0.03 (-0.01, 0.07)  0.01 (-0.03, 0.06)  **0.06 (0.02, 0.11)**  ***p=0.008*** | 0.00 [Reference]  0.01 (-0.08, 0.09)  0.00 (-0.09, 0.09)  0.01 (-0.08, 0.10)  *p=0.93* |  |
| DHQ cereal score, continuous | **0.004 (-0.004, 0.013)**  ***p=0.34*** | **0.002 (-0.009, 0.013)**  ***p=0.69*** | 0.010 (-0.007, 0.028)  *p=0.24* | 0.011 (-0.006, 0.029)  *p=0.21* | -0.000 (-0.036, 0.034)  *p=0.96* |  |
| <3   >3-4   >4-4.3   >4.3-5 *Trend* | 0.00 [Reference]  **0.02 (0.00, 0.04)**  0.01 (-0.01, 0.04)  0.02 (-0.01, 0.04)  *p=0.29* | 0.00 [Reference]  0.02 (-0.00, 0.05)  0.01 (-0.02, 0.04)  0.02 (-0.01, 0.05)  *p=0.15* | 0.00 [Reference]  0.03 (-0.01, 0.07)  0.04 (-0.00, 0.09)  0.02 (-0.02, 0.07)  *p=0.44* | 0.00 [Reference]  0.03 (-0.01, 0.07)  0.03 (-0.02, 0.08)  0.04 (-0.01, 0.08)  *p=0.13* | 0.00 [Reference]  0.01 (-0.07, 0.09)  0.02 (-0.08, 0.11)  0.01 (-0.08, 0.09)  *p=0.98* |  |
| DHQ fruit and vegetable score, continuous | **0.011 (0.002, 0.020)**  ***p=0.019*** | 0.005 (-0.008, 0.017)  *p=0.45* | 0.014 (-0.007, 0.035)  *p=0.19* | **0.024 (0.004, 0.043)**  ***p=0.020*** | -0.001 (-0.041, 0.038)  *p=0.95* |  |
| <3.2   >3.2-4   >4-4.4   >4.4-5 *Trend* | 0.00 [Reference]  0.01 (-0.01, 0.04)  0.01 (-0.01, 0.03)  **0.03 (0.00, 0.05)**  *p=0.058* | 0.00 [Reference]  0.00 (-0.02, 0.03)  0.02 (-0.01, 0.04)  0.01 (-0.02, 0.04)  *p=0.27* | 0.00 [Reference]  0.01 (-0.03, 0.05)  0.04 (-0.01, 0.08)  0.02 (-0.02, 0.07)  *p=0.28* | 0.00 [Reference]  0.03 (-0.01, 0.08)  **0.05 (0.00, 0.10)**  **0.05 (0.00, 0.10)**  ***p=0.025*** | 0.00 [Reference]  0.00 (-0.08, 0.09)  -0.01 (-0.10, 0.09)  -0.01 (-0.10, 0.09)  *p=0.93* |  |
| DHQ takeaway score, continuous | **0.010 (0.000, 0.020)**  ***p=0.047*** | 0.008 (-0.004, 0.021)  *p=0.18* | 0.003 (-0.017, 0.022)  *p=0.80* | 0.020 (-0.002, 0.042)  *p=0.068* | 0.006 (-0.033, 0.044)  *p=0.78* |  |
| <3.7   >3.7-4.3   >4.3-5 *Trend* | 0.00 [Reference]  -0.01 (-0.03, 0.01)  0.02 (-0.00, 0.03)  *p=0.065* | 0.00 [Reference]  0.01 (-0.02, 0.03)  0.01 (-0.01, 0.03)  *p=0.36* | 0.00 [Reference]  -0.03 (-0.07, 0.01)  -0.00 (-0.04, 0.03)  *p=0.74* | 0.00 [Reference]  0.02 (-0.03, 0.06)  0.04 (-0.00, 0.08)  *p=0.055* | 0.00 [Reference]  0.02 (-0.06, 0.10)  0.01 (-0.07, 0.08)  *p=0.93* |  |
| DHQ food choices score, continuous | 0.009 (-0.002, 0.019)  *p=0.096* | 0.009 (-0.004, 0.021)  *p=0.18* | 0.017 (-0.004, 0.037)  *p=0.11* | **0.029 (0.0009, 0.049)**  ***p=0.004*** | 0.001 (-0.038, 0.040)  *p=0.97* |  |
| <3.8   >3.8-4.5   >4.5-5 *Trend* | 0.00 [Reference]  0.01 (-0.01, 0.04)  0.02 (-0.00, 0.04)  *p=0.069* | 0.00 [Reference]  0.02 (-0.01, 0.04)  0.02 (-0.01, 0.05)  *p=0.20* | 0.00 [Reference]  0.02 (-0.02, 0.06)  0.04 (-0.00, 0.07)  *p=0.078* | 0.00 [Reference]  **0.04 (0.00, 0.09)**  **0.07 (0.03, 0.11)**  ***p=0.001*** | 0.00 [Reference]  0.00 (-0.08, 0.09)  0.00 (-0.08, 0.08)  *p=0.98* |  |
| DHQ omega-3 score, continuous | 0.004 (-0.002, 0.010)  *p=0.16* | 0.006 (-0.001, 0.013)  *p=0.11* | 0.003 (-0.009, 0.016)  *p=0.59* | **0.015 (0.003, 0.027)**  ***p=0.015*** | 0.001 (-0.023, 0.024)  *p=0.96* |  |
| <3   >3-4   >4-5  *Trend* | 0.00 [Reference]  -0.00 (-0.02, 0.02)  0.02 (-0.01, 0.04)  *p=0.076* | 0.00 [Reference]  -0.01 (-0.04, 0.01)  0.02 (-0.00, 0.04)  *p=0.10* | 0.00 [Reference]  -0.02 (-0.06, 0.02)  0.02 (-0.01, 0.06)  *p=0.34* | 0.00 [Reference]  0.01 (-0.03, 0.05)  0.04 (-0.00, 0.07)  *p=0.10* | 0.00 [Reference]  -0.02 (-0.10, 0.06)  0.00 (-0.07, 0.07)  *p=0.93* |  |
| DHQ food preparation score, continuous | 0.011 (-0.003, 0.025)  *p=0.11* | 0.001 (-0.018, 0.020)  *p=0.90* | 0.016 (-0.013, 0.045)  *p=0.28* | **0.031 (0.002, 0.060)**  ***p=0.037*** | 0.014 (-0.044, 0.072)  *p=0.64* |  |
| <4.4   >4.4-5 | 0.00 [Reference]  0.01 (-0.00, 0.03)  *p=0.10* | 0.00 [Reference]  0.01 (-0.02, 0.03)  *p=0.58* | 0.00 [Reference]  0.02 (-0.01, 0.06)  *p=0.20* | 0.00 [Reference]  **0.06 (0.02, 0.09)**  ***p=0.001*** | 0.00 [Reference]  0.01 (-0.06, 0.08)  *p=0.70* |  |
| DHQ fibre score, continuous | 0.011 (-0.000, 0.022)  *p=0.056* | 0.005 (-0.010, 0.020)  *p=0.51* | 0.013 (-0.009, 0.035)  *p=0.25* | **0.027 (0.004, 0.049)**  ***p=0.023*** | -0.001 (-0.039, 0.037)  *p=0.97* |  |
| <3.3   >3.3-3.9   >3.9-4.4   >4.4-5 *Trend* | 0.00 [Reference]  0.02 (-0.01, 0.04)  0.02 (-0.00, 0.04)  0.02 (-0.00, 0.04)  *p=0.088* | 0.00 [Reference]  -0.00 (-0.03, 0.03)  0.00 (-0.03, 0.03)  0.01 (-0.02, 0.04)  *p=0.53* | 0.00 [Reference]  -0.00 (-0.05, 0.04)  0.03 (-0.02, 0.07)  0.01 (-0.04, 0.06)  *p=0.53* | 0.00 [Reference]  0.04 (-0.00, 0.09)  0.03 (-0.02, 0.07)  0.04 (-0.01, 0.09)  *p=0.27* | 0.00 [Reference]  0.00 (-0.07, 0.08)  -0.00 (-0.08, 0.07)  -0.00 (-0.08, 0.08)  *p=0.96* |  |
| Consumes meat   No   Yes | 0.00 [Reference]  -0.01 (-0.03, 0.00)  *p=0.092* | 0.00 [Reference]  -0.01 (-0.03, 0.01)  *p=0.26* | 0.00 [Reference]  **-0.05 (-0.08, -0.02)**  ***p=0.001*** | 0.00 [Reference]  **-0.04 (-0.08, -0.01)**  ***p=0.011*** | 0.00 [Reference]  0.00 (-0.06, 0.06)  *p=0.99* |  |
| Consumes dairy   No    Yes | 0.00 [Reference]  **-0.02 (-0.03, -0.00)**  ***p=0.018*** | 0.00 [Reference]  **-0.03 (-0.05, -0.01)**  ***p=0.002*** | 0.00 [Reference]  -0.03 (-0.06, 0.00)  *p=0.068* | 0.00 [Reference]  **-0.04 (-0.07, -0.00)**  ***p=0.024*** | 0.00 [Reference]  0.00 (-0.06, 0.06)  *p=1.00* |  |
| SF-6D score | **Total HSU** | | **Social functioning** | | **Pain** | |
|  | aβ (95% CI) (2.5 years) | aβ (95% CI) (5 years) | aβ (95% CI) (2.5 years) | aβ (95% CI) (5 years) | aβ (95% CI) (2.5 years) | aβ (95% CI) (5 years) |
| DHQ total score, 10-unit continuous | **0.008 (0.001, 0.016)**  ***p=0.025*** | 0.005 (-0.005, 0.015)  *p=0.31* | 0.003 (-0.015, 0.021)  *p=0.73* | -0.009 (-0.032, 0.014)  *p=0.43* | **0.021 (0.004, 0.038)**  ***p=0.015*** | 0.013 (-0.003, 0.029)  *p=0.11* |
| <75   >75-85   >85-92   >92-100 *Trend* | 0.00 [Reference]  0.01 (-0.01, 0.04)  0.03 (-0.00, 0.05)  0.02 (-0.00, 0.05)  ***p=0.047*** | 0.00 [Reference]  0.01 (-0.02, 0.04)  0.01 (-0.02, 0.04)  0.03 (-0.00, 0.05)  *p=0.10* | 0.00 [Reference]  0.00 (-0.05, 0.05)  0.01 (-0.05, 0.06)  -0.00 (-0.06, 0.05)  *p=0.90* | 0.00 [Reference]  -0.01 (-0.07, 0.06)  -0.04 (-0.11, 0.03)  -0.02 (-0.09, 0.05)  *p=0.38* | 0.00 [Reference]  0.04 (-0.01, 0.09) **0.05 (-0.00, 0.10)**  **0.06 (0.01, 0.11)**  *p=0.056* | 0.00 [Reference]  0.03 (-0.01, 0.08)  0.04 (-0.01, 0.09)  **0.06 (0.01, 0.11)**  ***p=0.041*** |
| DHQ fat score, continuous | **0.015 (0.001, 0.028)**  ***p=0.033*** | 0.011 (-0.008, 0.029)  *p=0.26* | 0.012 (-0.023, 0.047)  *p=0.49* | -0.016 (-0.060, 0.029)  *p=0.49* | **0.044 (0.012, 0.076)**  ***p=0.007*** | **0.033 (0.000, 0.064)**  ***p=0.046*** |
| <3.7   >3.7-4.2   >4.2-4.7   >4.7-5 *Trend* | 0.00 [Reference]  **0.01 (-0.01, 0.03)**  0.01 (-0.01, 0.04)  **0.03 (0.01, 0.05)**  ***p=0.015*** | 0.00 [Reference]  0.01 (-0.02, 0.04)  -0.01 (-0.04, 0.02)  0.03 (-0.00, 0.05)  *p=0.16* | 0.00 [Reference]  0.03 (-0.02, 0.08)  0.02 (-0.04, 0.07)  0.03 (-0.03, 0.08)  *p=0.70* | 0.00 [Reference]  -0.02 (-0.09, 0.05)  -0.05 (-0.12, 0.02)  -0.02 (-0.08, 0.05)  *p=0.53* | 0.00 [Reference]  **0.09 (0.04, 0.13)**  **0.09 (0.04, 0.14)**  **0.08 (0.03, 0.13)**  ***p=0.033*** | 0.00 [Reference]  0.04 (-0.01, 0.09)  0.03 (-0.02, 0.08)  **0.07 (0.01, 0.12)**  *p=0.050* |
| DHQ cereal score, continuous | **0.004 (-0.004, 0.013)**  ***p=0.34*** | **0.002 (-0.009, 0.013)**  ***p=0.69*** | -0.001 (-0.021, 0.020)  *p=0.94* | -0.007 (-0.035, 0.020)  *p=0.60* | **0.020 (0.001, 0.040)**  ***p=0.044*** | 0.007 (-0.012, 0.026)  *p=0.49* |
| <3   >3-4   >4-4.3   >4.3-5 *Trend* | 0.00 [Reference]  **0.02 (0.00, 0.04)**  0.01 (-0.01, 0.04)  0.02 (-0.01, 0.04)  *p=0.29* | 0.00 [Reference]  0.02 (-0.00, 0.05)  0.01 (-0.02, 0.04)  0.02 (-0.01, 0.05)  *p=0.15* | 0.00 [Reference]  0.01 (-0.04, 0.06)  0.01 (-0.05, 0.07)  0.00 (-0.05, 0.06)  *p=0.96* | 0.00 [Reference]  0.00 (-0.06, 0.06)  -0.00 (-0.08, 0.07)  -0.02 (-0.09, 0.03)  *p=0.49* | 0.00 [Reference]  0.05 (-0.00, 0.09)  0.05 (-0.00, 0.11)  0.04 (-0.01, 0.10)  *p=0.17* | 0.00 [Reference]  0.04 (-0.00, 0.09)  0.03 (-0.03, 0.09)  0.05 (-0.01, 0.10)  *p=0.16* |
| DHQ fruit and vegetable score, continuous | **0.011 (0.002, 0.020)**  ***p=0.019*** | 0.005 (-0.008, 0.017)  *p=0.45* | 0.001 (-0.024, 0.025)  *p=0.95* | -0.002 (-0.033, 0.029)  *p=0.91* | 0.012 (-0.011, 0.035)  *p=0.30* | 0.010 (-0.011, 0.032)  *p=0.34* |
| <3.2   >3.2-4   >4-4.4   >4.4-5 *Trend* | 0.00 [Reference]  0.01 (-0.01, 0.04)  0.01 (-0.01, 0.03)  **0.03 (0.00, 0.05)**  *p=0.058* | 0.00 [Reference]  0.00 (-0.02, 0.03)  0.02 (-0.01, 0.04)  0.01 (-0.02, 0.04)  *p=0.27* | 0.00 [Reference]  **0.02 (-0.04, 0.07)**  -0.00 (-0.06, 0.05)  0.01 (-0.05, 0.07)  *p=0.91* | 0.00 [Reference]  -0.00 (-0.07, 0.06)  -0.02 (-0.09, 0.05)  -0.01 (-0.08, 0.07)  *p=0.93* | 0.00 [Reference]  -0.02 (-0.06, 0.03)  0.01 (-0.04, 0.06)  0.01 (-0.04, 0.06)  *p=0.34* | 0.00 [Reference]  0.02 (-0.03, 0.06)  0.04 (-0.01, 0.09)  0.01 (-0.04, 0.06)  *p=0.34* |
| DHQ takeaway score, continuous | **0.010 (0.000, 0.020)**  ***p=0.047*** | 0.008 (-0.004, 0.021)  *p=0.18* | -0.002 (-0.025, 0.022)  *p=0.88* | -0.001 (-0.025, 0.024)  *p=0.94* | 0.018 (-0.005, 0.040)  *p=0.13* | **0.026 (0.004, 0.047)**  ***p=0.018*** |
| <3.7   >3.7-4.3   >4.3-5 *Trend* | 0.00 [Reference]  -0.01 (-0.03, 0.01)  0.02 (-0.00, 0.03)  *p=0.065* | 0.00 [Reference]  0.01 (-0.02, 0.03)  0.01 (-0.01, 0.03)  *p=0.36* | 0.00 [Reference]  -0.00 (-0.05, 0.05)  -0.01 (-0.05, 0.04)  *p=0.70* | 0.00 [Reference]  0.01 (-0.04, 0.06)  -0.01 (-0.05, 0.04)  *p=0.81* | 0.00 [Reference]  0.03 (-0.02, 0.08)  0.03 (-0.02, 0.07)  *p=0.23* | 0.00 [Reference]  0.02 (-0.03, 0.07)  **0.04 (0.00, 0.08)**  ***p=0.037*** |
| DHQ food choices score, continuous | 0.009 (-0.002, 0.019)  *p=0.096* | 0.009 (-0.004, 0.021)  *p=0.18* | 0.015 (-0.009, 0.038)  *p=0.22* | -0.008 (-0.039, 0.022)  *p=0.60* | **0.033 (0.011, 0.055)**  ***p=0.004*** | 0.017 (-0.005, 0.040)  *p=0.13* |
| <3.8   >3.8-4.5   >4.5-5 *Trend* | 0.00 [Reference]  0.01 (-0.01, 0.04)  0.02 (-0.00, 0.04)  *p=0.069* | 0.00 [Reference]  0.02 (-0.01, 0.04)  0.02 (-0.01, 0.05)  *p=0.20* | 0.00 [Reference]  0.03 (-0.02, 0.08)  0.03 (-0.02, 0.06)  *p=0.35* | 0.00 [Reference]  0.00 (-0.07, 0.07)  -0.01 (-0.07, 0.05)  *p=0.70* | 0.00 [Reference]  0.04 (-0.00, 0.09)  **0.06 (0.01, 0.11)**  ***p=0.014*** | 0.00 [Reference]  0.01 (-0.03, 0.06)  0.04 (-0.00, 0.09)  ***p=0.041*** |
| DHQ omega-3 score, continuous | 0.004 (-0.002, 0.010)  *p=0.16* | 0.006 (-0.001, 0.013)  *p=0.11* | 0.004 (-0.010, 0.019)  *p=0.55* | -0.003 (-0.022, 0.015)  *p=0.72* | 0.012 (-0.003, 0.026)  *p=0.12* | 0.009 (-0.004, 0.023)  *p=0.16* |
| <3   >3-4   >4-5  *Trend* | 0.00 [Reference]  -0.00 (-0.02, 0.02)  0.02 (-0.01, 0.04)  *p=0.076* | 0.00 [Reference]  -0.01 (-0.04, 0.01)  0.02 (-0.00, 0.04)  *p=0.10* | 0.00 [Reference]  0.01 (-0.04, 0.06)  0.01 (-0.03, 0.06)  *p=0.65* | 0.00 [Reference]  -0.01 (-0.07, 0.05)  -0.01 (-0.07, 0.04)  *p=0.70* | 0.00 [Reference]  0.00 (-0.05, 0.05)  **0.04 (0.00, 0.09)**  *p=0.062* | 0.00 [Reference]  0.02 (-0.03, 0.07)  0.04 (-0.00, 0.08)  *p=0.067* |
| DHQ food preparation score, continuous | 0.011 (-0.003, 0.025)  *p=0.11* | 0.001 (-0.018, 0.020)  *p=0.90* | 0.001 (-0.033, 0.035)  *p=0.97* | -0.017 (-0.063, 0.029)  *p=0.47* | **0.038 (0.003, 0.072)**  ***p=0.034*** | 0.021 (-0.012, 0.054)  *p=0.21* |
| <4.4   >4.4-5 | 0.00 [Reference]  0.01 (-0.00, 0.03)  *p=0.10* | 0.00 [Reference]  0.01 (-0.02, 0.03)  *p=0.58* | 0.00 [Reference]  0.00 (-0.04, 0.05)  *p=0.84* | 0.00 [Reference]  -0.02 (-0.07, 0.04)  *p=0.56* | 0.00 [Reference]  **0.04 (0.00, 0.08)**  ***p=0.043*** | 0.00 [Reference]  0.02 (-0.02, 0.06)  *p=0.33* |
| DHQ fibre score, continuous | 0.011 (-0.000, 0.022)  *p=0.056* | 0.005 (-0.010, 0.020)  *p=0.51* | 0.001 (-0.027, 0.028)  *p=0.96* | -0.006 (-0.042, 0.030)  *p=0.74* | 0.021 (-0.004, 0.046)  *p=0.11* | 0.015 (-0.009, 0.039)  *p=0.22* |
| <3.3   >3.3-3.9   >3.9-4.4   >4.4-5 *Trend* | 0.00 [Reference]  0.02 (-0.01, 0.04)  0.02 (-0.00, 0.04)  0.02 (-0.00, 0.04)  *p=0.088* | 0.00 [Reference]  -0.00 (-0.03, 0.03)  0.00 (-0.03, 0.03)  0.01 (-0.02, 0.04)  *p=0.53* | 0.00 [Reference]  **0.01 (-0.05, 0.06)**  0.01 (-0.04, 0.07)  0.00 (-0.06, 0.06)  *p=0.96* | 0.00 [Reference]  0.01 (-0.06, 0.08)  -0.01 (-0.08, 0.06)  -0.01 (-0.08, 0.07)  *p=0.66* | 0.00 [Reference]  0.02 (-0.03, 0.07)  0.03 (-0.02, 0.08)  0.03 (-0.02, 0.08)  *p=0.18* | 0.00 [Reference]  0.02 (-0.03, 0.07)  0.03 (-0.02, 0.08)  0.03 (-0.02, 0.09)  *p=0.18* |
| Consumes meat   No   Yes | 0.00 [Reference]  -0.01 (-0.03, 0.00)  *p=0.092* | 0.00 [Reference]  -0.01 (-0.03, 0.01)  *p=0.26* | 0.00 [Reference]  0.01 (-0.03, 0.04)  *p=0.78* | 0.00 [Reference]  0.01 (-0.04, 0.06)  *p=0.70* | 0.00 [Reference]  -0.03 (-0.07, 0.00)  *p=0.082* | 0.00 [Reference]  **-0.05 (-0.08, -0.01)**  ***p=0.010*** |
| Consumes dairy   No    Yes | 0.00 [Reference]  **-0.02 (-0.03, -0.00)**  ***p=0.018*** | 0.00 [Reference]  **-0.03 (-0.05, -0.01)**  ***p=0.002*** | 0.00 [Reference]  -0.01 (-0.05, 0.03)  *p=0.59* | 0.00 [Reference]  -0.01 (-0.06, 0.05)  *p=0.83* | 0.00 [Reference]  -0.02 (-0.06, 0.02)  *p=0.30* | 0.00 [Reference]  -0.03 (-0.07, 0.01)  *p=0.093* |
| SF-6D score | **Total HSU** | | **Mental functioning** | | **Vitality** | |
|  | aβ (95% CI) (2.5 years) | aβ (95% CI) (5 years) | aβ (95% CI) (2.5 years) | aβ (95% CI) (5 years) | aβ (95% CI) (2.5 years) | aβ (95% CI) (5 years) |
| DHQ total score, 10-unit continuous | **0.008 (0.001, 0.016)**  ***p=0.025*** | 0.005 (-0.005, 0.015)  *p=0.31* |  | 0.007 (-0.009, 0.023)  *p=0.41* | **0.017 (0.000, 0.034)**  ***p=0.045*** | **0.020 (0.005, 0.035)**  ***p=0.010*** |
| <75   >75-85   >85-92   >92-100 *Trend* | 0.00 [Reference]  0.01 (-0.01, 0.04)  0.03 (-0.00, 0.05)  0.02 (-0.00, 0.05)  ***p=0.047*** | 0.00 [Reference]  0.01 (-0.02, 0.04)  0.01 (-0.02, 0.04)  0.03 (-0.00, 0.05)  *p=0.10* |  | 0.00 [Reference]  -0.01 (-0.05, 0.04)  0.01 (-0.04, 0.06)  0.02 (-0.03, 0.07)  *p=0.42* | 0.00 [Reference]  0.03 (-0.02, 0.08)  0.04 (-0.01, 0.09)  **0.07 (0.02, 0.12)**  ***p=0.010*** | 0.00 [Reference]  0.03 (-0.02, 0.07)  0.04 (-0.00, 0.09)  **0.07 (0.02, 0.11)**  ***p=0.004*** |
| DHQ fat score, continuous | 0.015 (0.001, 0.028)  *p=0.033* | 0.011 (-0.008, 0.029)  *p=0.26* |  | 0.006 (-0.017, 0.030)  *p=0.59* | 0.013 (-0.020, 0.045)  *p=0.43* | **0.041 (0.012, 0.069)**  ***p=0.006*** |
| <3.7   >3.7-4.2   >4.2-4.7   >4.7-5 *Trend* | 0.00 [Reference]  **0.01 (-0.01, 0.03)**  0.01 (-0.01, 0.04)  **0.03 (0.01, 0.05)**  ***p=0.015*** | 0.00 [Reference]  0.01 (-0.02, 0.04)  -0.01 (-0.04, 0.02)  0.03 (-0.00, 0.05)  *p=0.16* |  | 0.00 [Reference]  -0.01 (-0.06, 0.03)  -0.01 (-0.06, 0.04)  0.02 (-0.03, 0.06)  *p=0.50* | 0.00 [Reference]  0.01 (-0.04, 0.06)  -0.01 (-0.07, 0.04)  0.05 (-0.00, 0.10)  *p=0.14* | 0.00 [Reference]  0.03 (-0.01, 0.08)  0.02 (-0.03, 0.06)  **0.06 (0.02, 0.11)**  ***p=0.015*** |
| DHQ cereal score, continuous | **0.004 (-0.004, 0.013)**  ***p=0.34*** | **0.002 (-0.009, 0.013)**  ***p=0.69*** |  | 0.012 (-0.006, 0.031)  *p=0.20* | 0.010 (-0.011, 0.031)  *p=0.34* | 0.004 (-0.015, 0.022)  *p=0.71* |
| <3   >3-4   >4-4.3   >4.3-5 *Trend* | 0.00 [Reference]  **0.02 (0.00, 0.04)**  0.01 (-0.01, 0.04)  0.02 (-0.01, 0.04)  *p=0.29* | 0.00 [Reference]  0.02 (-0.00, 0.05)  0.01 (-0.02, 0.04)  0.02 (-0.01, 0.05)  *p=0.15* |  | 0.00 [Reference]  0.01 (-0.04, 0.05)  0.01 (-0.04, 0.07)  0.02 (-0.03, 0.07)  *p=0.39* | 0.00 [Reference]  0.02 (-0.03, 0.07)  0.03 (-0.03, 0.09)  0.03 (-0.02, 0.09)  *p=0.15* | 0.00 [Reference]  0.04 (-0.01, 0.08)  0.02 (-0.04, 0.08)  0.02 (-0.03, 0.07)  *p=0.69* |
| DHQ fruit and vegetable score, continuous | **0.011 (0.002, 0.020)**  ***p=0.019*** | 0.005 (-0.008, 0.017)  *p=0.45* |  | 0.007 (-0.014, 0.029)  *p=0.51* | **0.032 (0.007, 0.056)**  ***p=0.012*** | **0.034 (0.015, 0.053)**  ***p=0.001*** |
| <3.2   >3.2-4   >4-4.4   >4.4-5 *Trend* | 0.00 [Reference]  0.01 (-0.01, 0.04)  0.01 (-0.01, 0.03)  **0.03 (0.00, 0.05)**  *p=0.058* | 0.00 [Reference]  0.00 (-0.02, 0.03)  0.02 (-0.01, 0.04)  0.01 (-0.02, 0.04)  *p=0.27* |  | 0.00 [Reference]  0.01 (-0.04, 0.05)  0.01 (-0.03, 0.06)  0.01 (-0.04, 0.06)  *p=0.48* | 0.00 [Reference]  0.03 (-0.01, 0.08)  0.05 (-0.01, 0.10)  **0.10 (0.04, 0.15)**  ***p=0.001*** | 0.00 [Reference]  0.04 (-0.00, 0.08)  **0.06 (0.01, 0.10)**  **0.08 (0.03, 0.12)**  ***p<0.001*** |
| DHQ takeaway score, continuous | **0.010 (0.000, 0.020)**  ***p=0.047*** | 0.008 (-0.004, 0.021)  *p=0.18* |  | 0.009 (-0.011, 0.029)  *p=0.39* | 0.020 (-0.003, 0.043)  *p=0.085* | **0.020 (0.000, 0.040)**  ***p=0.047*** |
| <3.7   >3.7-4.3   >4.3-5 *Trend* | 0.00 [Reference]  -0.01 (-0.03, 0.01)  0.02 (-0.00, 0.03)  *p=0.065* | 0.00 [Reference]  0.01 (-0.02, 0.03)  0.01 (-0.01, 0.03)  *p=0.36* |  | 0.00 [Reference]  0.01 (-0.04, 0.05)  0.01 (-0.03, 0.05)  *p=0.47* | 0.00 [Reference]  0.02 (-0.03, 0.08)  0.03 (-0.02, 0.07)  *p=0.22* | 0.00 [Reference]  0.01 (-0.03, 0.06)  0.04 (-0.00, 0.08)  ***p=0.030*** |
| DHQ food choices score, continuous | 0.009 (-0.002, 0.019)  *p=0.096* | 0.009 (-0.004, 0.021)  *p=0.18* |  | -0.005 (-0.021, 0.012)  *p=0.56* | 0.010 (-0.012, 0.032)  *p=0.39* | 0.020 (-0.001, 0.041)  *p=0.059* |
| <3.8   >3.8-4.5   >4.5-5 *Trend* | 0.00 [Reference]  0.01 (-0.01, 0.04)  0.02 (-0.00, 0.04)  *p=0.069* | 0.00 [Reference]  0.02 (-0.01, 0.04)  0.02 (-0.01, 0.05)  *p=0.20* |  | 0.00 [Reference]  -0.02 (-0.06, 0.03)  -0.01 (-0.05, 0.03)  *p=0.50* | 0.00 [Reference]  -0.02 (-0.07, 0.02)  0.04 (-0.01, 0.08)  *p=0.081* | 0.00 [Reference]  0.00 (-0.05, 0.05)  0.04 (-0.01, 0.08)  *p=0.058* |
| DHQ omega-3 score, continuous | 0.004 (-0.002, 0.010)  *p=0.16* | 0.006 (-0.001, 0.013)  *p=0.11* |  | -0.002 (-0.012, 0.007)  *p=0.62* | 0.007 (-0.007, 0.021)  *p=0.30* | 0.010 (-0.003, 0.023)  *p=0.12* |
| <3   >3-4   >4-5  *Trend* | 0.00 [Reference]  -0.00 (-0.02, 0.02)  0.02 (-0.01, 0.04)  *p=0.076* | 0.00 [Reference]  -0.01 (-0.04, 0.01)  0.02 (-0.00, 0.04)  *p=0.10* |  | 0.00 [Reference]  -0.02 (-0.07, 0.02)  -0.00 (-0.04, 0.04)  *p=0.94* | 0.00 [Reference]  0.00 (-0.05, 0.05)  0.01 (-0.03, 0.06)  *p=0.46* | 0.00 [Reference]  0.01 (-0.03, 0.06)  0.04 (-0.00, 0.08)  *p=0.059* |
| DHQ food preparation score, continuous | **0.011 (-0.003, 0.025)**  ***p=0.11*** | 0.001 (-0.018, 0.020)  *p=0.90* |  | 0.018 (-0.006, 0.041)  *p=0.14* | -0.010 (-0.044, 0.024)  *p=0.55* | **0.031 (0.002, 0.060)**  ***p=0.038*** |
| <4.4   >4.4-5 | 0.00 [Reference]  0.01 (-0.00, 0.03)  *p=0.10* | 0.00 [Reference]  0.01 (-0.02, 0.03)  *p=0.58* |  | 0.00 [Reference]  0.01 (-0.02, 0.04)  *p=0.57* | 0.00 [Reference]  0.00 (-0.04, 0.05)  *p=0.85* | 0.00 [Reference]  **0.04 (0.00, 0.08)**  ***p=0.029*** |
| DHQ fibre score, continuous | 0.011 (-0.000, 0.022)  *p=0.056* | 0.005 (-0.010, 0.020)  *p=0.51* |  | **0.018 (0.000, 0.036)**  ***p=0.049*** | **0.033 (0.005, 0.061)**  ***p=0.020*** | **0.033 (0.010, 0.056)**  ***p=0.005*** |
| <3.3   >3.3-3.9   >3.9-4.4   >4.4-5 *Trend* | 0.00 [Reference]  0.02 (-0.01, 0.04)  0.02 (-0.00, 0.04)  0.02 (-0.00, 0.04)  *p=0.088* | 0.00 [Reference]  -0.00 (-0.03, 0.03)  0.00 (-0.03, 0.03)  0.01 (-0.02, 0.04)  *p=0.53* |  | 0.00 [Reference]  -0.00 (-0.05, 0.04)  0.01 (-0.03, 0.05)  0.03 (-0.02, 0.07)  *p=0.12* | 0.00 [Reference]  0.04 (-0.01, 0.09)  0.03 (-0.03, 0.08)  **0.06 (0.01, 0.12)**  ***p=0.037*** | 0.00 [Reference]  0.05 (0.01, 0.09)  0.01 (-0.03, 0.06)  **0.06 (0.02, 0.11)**  ***p=0.024*** |
| Consumes meat   No   Yes | 0.00 [Reference]  -0.01 (-0.03, 0.00)  *p=0.092* | 0.00 [Reference]  -0.01 (-0.03, 0.01)  *p=0.26* |  | 0.00 [Reference]  0.02 (-0.01, 0.04)  *p=0.24* | 0.00 [Reference]  -0.01 (-0.05, 0.03)  *p=0.58* | 0.00 [Reference]  -0.01 (-0.05, 0.02)  *p=0.45* |
| Consumes dairy   No    Yes | 0.00 [Reference]  **-0.02 (-0.03, -0.00)**  ***p=0.018*** | 0.00 [Reference]  **-0.03 (-0.05, -0.01)**  ***p=0.002*** |  | 0.00 [Reference]  0.00 (-0.03, 0.03)  *p=0.98* | 0.00 [Reference]  -0.04 (-0.07, 0.00)  *p=0.053* | 0.00 [Reference]  -0.03 (-0.06, 0.01)  *p=0.097* |
| Abbreviations: aRR: adjusted Relative Risk; DHQ: Dietary Habits Questionnaire; HSU: Health State Utility  Analyses by quantile regression, estimating adjusted difference in median (β) (95% CI).  Note: Boldface denotes significance (p<0.05). Shaded cells indicate where quantitative analysis was not possible.  Model adjusted for experiencing ongoing symptoms due to relapse, age, sex, level of highest education, disability (P-MSSS), clinically significant fatigued, prescription antidepressant medication use, treated comorbidity number, depression-risk (PHQ-2), and baseline HSU. | | | | | | |
